# Holistic analysis of urban water systems in the Greater Cincinnati region: (1) life cycle assessment and cost implications

**DOI:** 10.1016/j.wroa.2018.100015

**Published:** 2018-12-14

**Authors:** Xiaobo Xue, Sarah Cashman, Anthony Gaglione, Janet Mosley, Lori Weiss, Xin Cissy Ma, Jennifer Cashdollar, Jay Garland

**Affiliations:** a Department of Environmental Health Sciences, School of Public Health, State University of New York at Albany, 1 University Drive, Rensselaer, NY, 12144, USA; b Eastern Research Group, Inc. (ERG), 110 Hartwell Avenue, Lexington, MA, 02421, USA; c National Risk Management Research Laboratory, U.S. Environmental Protection Agency Office of Research and Development, 26 W Martin Luther King Drive, Cincinnati, OH, 45268, USA; d National Exposure Research Laboratory, U.S. Environmental Protection Agency, Office of Research and Development, 26 W Martin Luther King Drive, Cincinnati, OH, 45268, USA

**Keywords:** Water, Wastewater, Urban water systems, Life cycle assessment, Environmental impacts, Cost

## Abstract

Urban water and wastewater utilities are striving to improve their environmental and economic performances due to multiple challenges such as increasingly stringent quality criterion, aging infrastructure, constraining financial burden, growing urban population, climate challenges and dwindling resources. Growing needs of holistic assessments of urban water systems are required to identify systems-level cross-domain solutions. This study evaluated the life cycle environmental and economic impacts of urban water and wastewater systems with two utilities in Greater Cincinnati region as a case study. The scope of this study includes the entire urban water and wastewater systems starting from raw water acquisition for drinking water to wastewater treatment and discharge. The detailed process-based life cycle models were developed based on the datasets provided by local water and wastewater utilities. The life cycle assessment indicated that the operation and maintenance of drinking water distribution was a dominating contributor for energy consumption (43%) and global warming potential (41%). Wastewater discharge from the wastewater treatment plant contributed to more than 80% of the total eutrophication potential. The cost analysis determined that labor and maintenance cost (19%) for wastewater collection, and electricity cost (13%) for drinking water distribution were major contributors. Electricity purchased by the utility was the driver for the majority of impact categories assessed with the exception of eutrophication, blue water use, and metal depletion. Infrastructure requirements had a negligible influence on impact results, contributing less than 3% to most categories, with the exception of metal depletion where it led to 68% of total burdens. Sensitivity analysis showed that the life cycle environmental results were more sensitive to the choice of the electricity mixes and electricity consumption than the rest of input parameters such as chemical dosages, and infrastructure life time. This is one of the first comprehensive studies of the whole urban water system using real case data. It elucidates a bigger picture of energy, resource and cost distributions in a typical urban centralized water system. Inherent to a modern city as large population centers, a significant expenditure has to be invested to provide water services function (moving water, treating water/wastewater) in order to avoid human and environmental health problems. This study provides insights for optimization potentials of overall treatment efficiency and can serve as a benchmark for communities considering adoption of alternative water systems.

## Highlights

• Process-based LCA models reveals expenditure of all stages in an urban water system.
• Drinking water distribution is the major contributor to environmental impacts.
• The operational stages contribute to most environmental impact categories.
• The operational cost is sensitive to electricity mix, unit price and consumption.
• The LCA and LCC framework aids long-term balanced decision making.

## 1 Introduction

Sustainable urban water systems are vital to provide the public with drinking water and sanitation services, and to promote the balanced environmental, economic, and social health of communities now and in the future. Currently, most urban water systems in developed countries have been managed to satisfy the urban water and sanitation demands through centralized configurations. However, with multiple challenges such as increasingly stringent quality criterion, aging infrastructure, constraining financial burden, growing urban population, climate effects and the promotion of sustainable and healthy communities, there is urgent need for systematically evaluating the performance of the current infrastructure and identifying strategies to improve the efficiency and sustainability of urban water system management.

Life cycle assessment (LCA) and Life cycle cost (LCC) analysis can assess the whole urban water system in a comprehensive way to identify critical processes and potential areas for improvement of the system. LCA is a well-established system accounting method to quantify energy consumption and environmental impacts through the entire life-cycle of a product or process. LCA studies of water systems track various environmental impacts derived from direct and supply chain activities. Complementary to the LCA approach, LCC analyses quantify the financial costs of water systems from raw material extraction, construction, operation, and demolishment at the end of life.

The existing body of literature contributes greatly to the understanding of the environmental and economic impacts of the whole urban water system, but lack in the following three areas: First, life cycle studies of whole urban water systems in the U.S., based on the use of real utility datasets, are still missing. Life cycle studies of whole urban water systems are necessary to understand the relative contributions of water and wastewater systems, and to serve as a baseline assessment for system optimization and adaptation (Loubet et al., 2014; Ma et al., 2015; Xue et al., 2015). As documented by a recent review article (Loubet et al., 2014) and several recent case studies (Jeong et al., 2015; Lane et al., 2015; Xue et al., 2016), a few LCA studies quantified the life cycle environmental impacts of the entire urban water and wastewater system (Amores et al., 2013, Arpke and Hutzler, 2006, Jeong et al., 2015; Lane et al., 2015; Lemos et al., 2013; Loubet et al., 2014; Lundie et al., 2004, Mahgoub et al., 2010, Slagstad and Brattebø, 2014). The majority of these studies have focused on water and wastewater services in Europe and Australia, with some exceptions (Arpke and Hutzler, 2006; Jeong et al., 2015; Xue et al., 2016). It was estimated the energy consumption and global warming potential (GWP) of a water and wastewater system in the United States without cost implications (Arpke and Hutzler, 2006). The life cycle environmental impacts of water and wastewater systems in Atlanta was assessed (Jeong et al., 2015), but the study heavily relied on European datasets for the infrastructure construction phase. Although another study (Xue et al. 2016) evaluated the impacts of various water and wastewater option, the scales were at the household level. There are differences in unit treatment processes in U.S. and other areas in the world such as disinfection or disposal strategies. The LCA of urban water and wastewater treatment built with actual utility datasets in North America are still needed to understand how the local specificity could affect the outcome and how the results can be used for decision making.

Another limitation is that many LCA studies of whole urban water systems have not identified the contribution of the unit processes to the overall life cycle environmental impacts. Although the aggregation of the processes might be helpful as a screening approach, it is not particularly useful for decision makers pinpointing the issues and finding solutions. A few studies have assessed the contribution of unit processes in either water or wastewater treatment plants. However, the scope of these studies (Bonton and Barbeau, 2012; Hospido et al., 2010; Igos et al., 2014; Langevin et al., 2010; Lederer and Rechberger, 2010; Pradel et al., 2010) are limited to a single or a few stages of the whole urban system. The analyses that cover the entire water system and assess stage contributions at unit process level to the environmental impacts of the overall water system remain limited. Lastly, the debate of relative contributions between infrastructure and operational phases is ongoing. Some found that the contributions of construction phase were minimal while others indicated a significant role in multiple environmental categories. Detailed inventories of infrastructure materials and machinery, and associated energy requirements, are necessary to elucidate the relative contributions of infrastructure phase.

Expanding on previous work, this study presents a life cycle environmental and economic analysis of an urban water system as a whole using U.S. specific real-case data from the Greater Cincinnati region. This study developed more detailed inventory and assessment in two major areas including 1) the contribution of unit processes in the entire water and wastewater treatment trains, and 2) the contribution of infrastructure stage at the unit process level. A companion emergy study (Arden et al., 2019) was also conducted to evaluate the same system from the thermodynamic perspective for a multi-faceted evaluation. Together they provide a more complete picture of energy, resource and cost distributions in a typical urban water system while providing insights for more sustainable water system management.

## 2 Method

### 2.1 Life cycle assessment

Standard approaches for goal and scope definition, inventory analysis, impact analysis, and interpretation as described by the International Organization for Standardization's (ISO) 14040 series were used and summarized below (International Organization for Standardization, 2010).

#### 2.1.1 Goal and scope

**Fig. 1:**
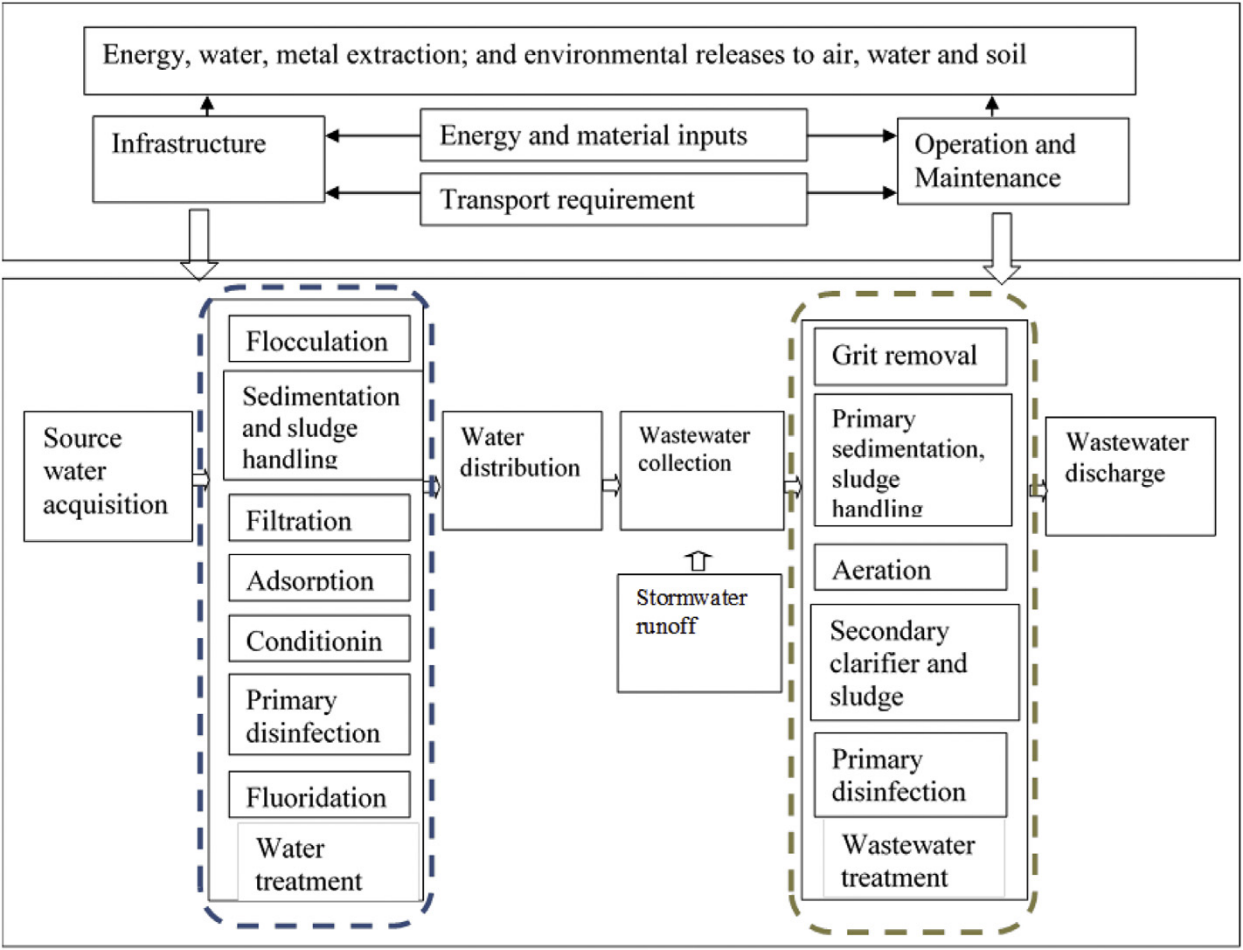
Water and wastewater systems in Cincinnati and the associated life cycle system boundary.

This study aims to understand 1) the detailed process-based life cycle models from real treatment plant data so that the utility managers are able to make targeted decisions; 2) the importance of integration of water and wastewater management and a bigger picture of energy, resource and cost distributions in a typical urban centralized water system; 3) the environmental impacts and costs of an urban water system using the Greater Cincinnati region as a case study, and identify the contributions of the unit processes on their respective impacts. Fig. 1 illustrates the system boundary of the LCA model. The processes start at the acquisition of source water from the Ohio River and end at the discharge of wastewater effluent to Mill Creek. Material, energy, and transportation inputs and environmental releases, which occurred during infrastructure construction and facility operation were included.

The 22 unit processes of water and wastewater systems were created to represent Richard Miller Drinking Water Treatment (DWT) and Mill Creek Wastewater Treatment Plants (WWTP) in Cincinnati, Ohio for the year 2011. Richard Miller DWT provides drinking water to parts of Hamilton, Butler, Warren, and Clermont Counties in Ohio and Boone County, Kentucky, with an operating capacity of 120 MGD. Mill Creek WWTP is the largest wastewater treatment plant among the 7 main plants within the Metropolitan Sewer District of Greater Cincinnati (MSDGC), with a nominal capacity of 120 MGD and maximum capacity of 360 MGD for combined sewer during wet weather. The unit processes for the water treatment train include source water acquisition, flocculation, sedimentation, filtration, adsorption, conditioning, primary disinfection, fluoridation, and water distribution. The unit processes for wastewater treatment train include wastewater collection, grit removal, primary sedimentation, aeration, secondary clarifier and sludge handling, primary disinfection and wastewater discharge. The detailed description of DWT and WWTP system is shown in Figs. S1 and S2.

The functional unit reflects that the provision of drinking water and sanitation services is the key service of water and wastewater systems. Aligned with previous LCA studies, 1 m^3^ of treated and distributed water meeting or exceeding National Primary Drinking Water Regulations was initially used as the functional unit for the drinking water analysis and 1 m^3^ of treated and discharged wastewater was initially used as the functional unit for the wastewater analysis. Results for drinking water and wastewater are combined here to a functional unit of 1 m^3^ of delivered drinking water, which is subsequently treated after use, to illustrate the comprehensive impact of municipal water treatment services. While on average, 30% of drinking water is used for outdoor activities in the US, this study assumes that in the long-term all drinking water will eventually be treated as wastewater, and the ratio of delivered drinking water to treated wastewater is 1:1 (Ma et al., 2015; National Research Council of the National Academies, 2006). Based on local utility datasets, 19% of treated drinking water was lost through leakage during distribution. Drinking water treatment plant (DWTP) impacts were, therefore, scaled up based on the distribution leakage rate. With the existing combined sewer system in Cincinnati, stormwater penetration accounts for 24% of flow to the WWTP during wet events. However, when the combined sewer flow exceeds the maximum capacity of the WWTP, the excess flow, presenting approximately 17% of the average annual flow, bypasses the secondary treatment. Since the majority of stormwater bypasses secondary treatment, the functional unit in this analysis is not adjusted based on stormwater flow, which can also vary greatly seasonally and from dry years to wet years.

#### 2.1.2 Life cycle inventory

The foreground inventory data for the life cycle inventory (LCI) were provided by Greater Cincinnati Water Works (GCWW) and the MSDGC through an iterative questionnaire process, and were documented using Federal Commons LCI Unit Process Templates (USDA National Agricultural Library, 2017). Additionally, site-visit and periodic discussions with utilities were conducted to ensure appropriate use of infrastructure and operational datasets. The detailed material, energy, and transport inputs for constructing and operating water and wastewater systems at unit process level are documented in Tables S1–S14. Since the year 2011 was the most recent year when the operational data for the water system was available for this study, the ultraviolet (UV) operation adopted by GCWW in 2013 was not included in the system boundary. For upstream processes such as chemical and electricity production, data were taken from the National Renewable Energy Laboratory (NREL) U.S. Life Cycle Inventory Database (US LCI) (National Renewable Energy Laboratory, 2012), a publicly available LCI source. The openLCA1.6 software was used to construct the LCI for the urban water system in Cincinnati due to its transparency and public availability.

In the WWTP, the biogenic carbon dioxide (CO_2_) emission from aeration and greenhouse gas (GHG) emissions from the sludge incineration process were computed for a complete GHG inventory (Table S8), but only the fossil CO_2_ was included in the impact assessment because municipal wastewater mostly comes from food, which has a short rotation period resulting in negligible net GWP (Cherubini et al., 2011; Liu et al., 2017). MSDGC provided the input parameters including volume of aerobic reactor, annual flow of influent wastewater, influent and effluent total suspended solids, and solids retention time, while the remaining parameters of a typical conventional activated sludge treatment system were obtained from literature (Monteith et al., 2005). The methane (CH_4_) and nitrous oxide (N_2_O) emissions from the aeration and sludge thickening processes were determined to be minimal (Foley and Lant, 2010). The GHG emissions of sludge incineration were estimated based on the Intergovernmental Panel on Climate Change (IPCC) 2006 guidelines, which included sludge volume and incineration temperature. According to the IPCC guidelines, a range of 40–50% of carbon content of dry sludge, and 4.85×10^−5^ kg of CH_4_ emitted/kg of dry sludge burned were used to estimate CH_4_ emissions (Intergovernmental Panel on Climate Change). N_2_O emissions were calculated based on a default value for nitrogen content of dry sludge published by the Biosolids Emissions Assessment Model (Canadian Council of Ministers of the Environment, 2009).

#### 2.1.3 Life cycle impact assessment

The Tool for the Reduction and Assessment of Chemical and Environmental Impacts (TRACI), version 2.0, developed by the U.S. EPA specifically to model environmental and human health impacts in the U.S., was the primary life cycle impact assessment (LCIA) method applied in this work (US Environmental Protection Agency, 2014). Additionally, the ReCiPe method was used to characterize fossil fuel and metal depletion (Goedkoop et al., 2008). Life cycle energy consumption was tracked using the cumulative energy demand method (Swiss Centre for Life Cycle Inventories, 2010). As guided by the water footprint assessment manual (Goedkoop et al., 2009), the blue water footprint served as an additional impact category to represent the direct and indirect water withdrawal in water and wastewater processes. The impact assessment categories and the underlying methods that were considered are summarized in Table S15. Life cycle impacts were analyzed to include the contributions of 1) various life cycle stages and unit processes within the entire treatment train, and 2) infrastructure and operation stages.

### 2.2 Life cycle cost assessment

The annual operational and maintenance (O&M) costs in year 2011 provided by GCWW were allocated to each unit process and normalized to 1 m^3^ of drinking water delivered for the water system. For the cost in wastewater, the annual O&M cost in year 2012 was provided by MSDGC and allocated to each unit process and normalized to 1 m^3^ of wastewater treated for the wastewater system. Delivered drinking water and wastewater life cycle costs were also combined to represent the same functional unit as the LCA. Plant-wide costs, such as insurance, and O&M labor costs, were calculated and represented in the overhead category. The infrastructure cost was not included in this study due to data limitation, since some pipes, tanks and machinery were more than a century old. Baseline drinking water and wastewater life cycle costs are important to incorporate as an additional metric in the analysis to understand trade-offs between unit process cost considerations and LCA findings. Baseline cost results can also be utilized in the future to understand how changing the treatment plant configurations and/or operations will impact the underlying economics of municipal water treatment.

### 2.3 Sensitivity analysis

Sensitivity analysis was performed to determine the influences of 16 key input parameters on the LCA and LCC results. The assessed input parameters included chlorine usage by the DWTP, lime usage by DWTP, alum coagulant usage by DWTP, sodium hypochlorite usages by DWTP and WWTP, natural gas usage for granular activated carbon (GAC) reactivation by DWTP, electricity usages at the DWTP, water distribution, wastewater collection and WWTP, respectively, carbon content of incinerated sludge, and lifetimes of DWTP, water distribution infrastructure, wastewater collection infrastructure and WWTP, respectively. In addition, the influences of choices of electricity grids on life cycle environmental impacts of the urban water system were assessed.

## 3 Results

### 3.1 Contribution of unit processes to the life cycle environmental and economic impacts

**Fig. 2:**
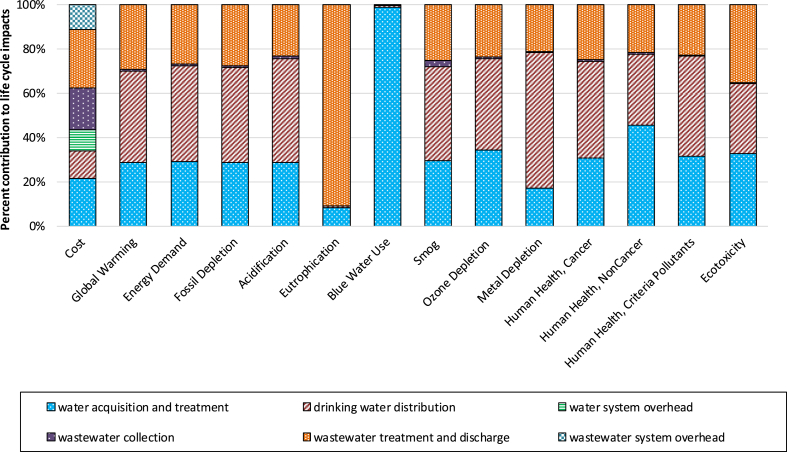
Process Contributions for Life Cycle Impacts of Water and Wastewater Systems in Cincinnati. Note: the overhead is only considered as a stage for cost. Cost analysis includes the costs during operation and maintenance stage.

The process contribution analysis (Fig. 2) indicates that electricity used for water distribution was the primary contributor for environmental impact categories including fossil fuel depletion and energy demand. The electricity consumption during DWTP in-plant pumping, water distribution and wastewater aeration resulted in 21%, 43%, and 13% of total fossil fuel depletion, respectively. Except for the gravity-driven wastewater collection system, moving water, whether in the network or in-plant, as well as disintegrating organics in wastewater, is energy-intensive (Stokes and Horvath, 2009). Detailed contribution results for specific unit processes in the life cycle of 1 m^3^ of water delivered and subsequently treated are provided in Table S17.

Moreover, water distribution (41%) and DWTP in-plant pumping (20%) ranked as the top two significant contributing processes for GWP. Other GWP contributors included aeration at the WWTP (12%) and sludge incineration (7%). Eutrophication impacts were overwhelmingly dominated by the release of wastewater effluent, even though the average ammonia (7.66 g/m^3^) and phosphorus (0.55 g/m^3^) levels in 2011 met the discharge standards. The second biggest contributor for eutrophication potential (5.5%) was the landfill disposal of sediment from the DWTP. The findings on eutrophication are consistent with previous studies (Amores et al., 2013; Buckley et al., 2011; Friedrich et al., 2009; Lassaux et al., 2007; Lundie et al., 2004, Mahgoub et al., 2010), which confirmed that nitrogen and phosphorus releases from the wastewater discharge were the major contributors to the eutrophication impact in other regions.

Furthermore, water distribution accounts for over 3,100 miles of piping (the collection system has less than 1,700 miles), and was the largest contributor (61%) to metal depletion. While the primary material for drinking water distribution network is ductile iron, the wastewater collection pipe network mainly consists of concrete pipes. Notably, productions of the chemicals used in water conditioning in DWTP to adjust pH added another 11% to the metal depletion results.

Also, two of the biggest contributors for human health noncancer impact included water distribution and chemical usage, together contributing to 65% of human health noncancer impact. While water distribution remained the biggest contributor (32%) to ecotoxicity impact, the second biggest contributor was sludge thickening and dewatering stage due to the application of polymer polyacrylamide and its upstream production.

In addition, cost based rankings were different from the life cycle impact rankings due to the inclusion of labor expenditure in cost. The LCC analysis found that wastewater collection accounted for 19% of the total cost mainly due to the expensive labor, followed by drinking water distribution contributing for 13% (Fig. 2). Sludge thickening, wastewater system overhead, and drinking water plant-wide overhead contributed to around 10–11% of the total cost. The rest of the stages contributed to less than 8% of the total cost.

### 3.2 Underlying drivers of life cycle environmental impacts

**Fig. 3:**
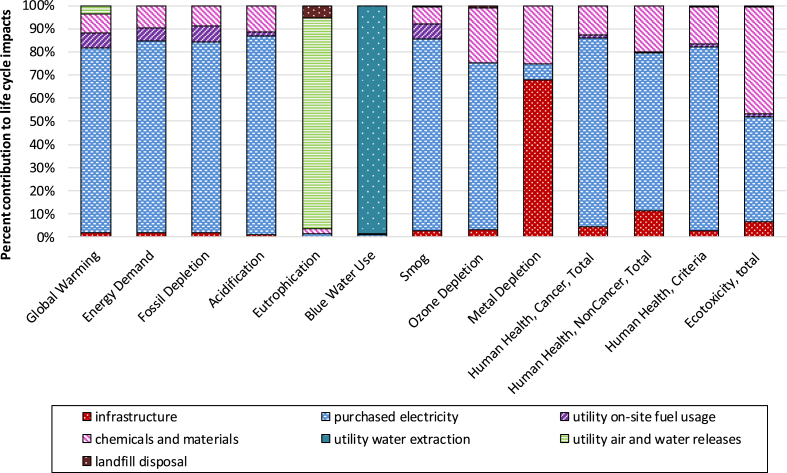
Contributions of underlying drivers to life cycle impacts of water and wastewater systems in Cincinnati.

Fig. 3 displays the underlying drivers of the life cycle impacts including direct on-site air and water releases as well as on-site fuel consumption, impacts associated with generation and delivery of electricity purchased by the utilities, burdens for infrastructure production, and impacts from chemical and material production and transportation. Upstream electricity generation and delivery processes from electricity purchased by the utilities were the primary contributors for global warming, energy demand, fossil depletion, acidification, smog, ozone depletion, and human health categories. The detailed unit process contributions to the life cycle impacts are list in Table S17.

As shown in Fig. 3, the infrastructure stage (combined water and wastewater) contributed to less than 10% of environmental impacts with the exception of metal depletion and human noncancer impact categories. The infrastructure stage dominated the metal depletion impact category (mainly due to the metal requirement for drinking water distribution and sewer collection networks). Infrastructure contributed 13% to total human health noncancer impact due to carbon disulfide air emissions from cast iron production processes (Table S18. The direct use of natural gas, diesel, and gasoline within water and wastewater treatment plants (categorized as utility on-site fuel usage) only resulted in 5% of life cycle energy demand. CH_4_ and NO_2_ directly generated during wastewater sludge incineration at the WWTP contributed to 3.5% of life cycle GWP. In contrast, eutrophication and blue water use were driven by on-site nutrient discharge from WWTP and water withdrawal of the DWTP, respectively. Additionally, cast iron use in piping networks during drinking water distribution and sewer collection contributed to 34% of life cycle metal depletion. Upstream impacts associated with purchased chemicals and materials contributed 7–20% to the majority of impacts, with the exception of eutrophication potential, blue water use and ecotoxicity. Chemical production also contributed 46% to ecotoxicty impacts, largely from acetic acid emission related to the sludge thickener polymer production as well as phenol, benzene and toluene emissions from upstream natural gas extraction for chemical use. The top contributing unit process and pollutants are summarized in Tables S19 and S20.

Our estimates of the infrastructure's contribution to the environmental impacts of the whole urban water system in Cincinnati were much lower than the estimates of Atlanta water system (Jeong et al., 2015). Found that infrastructure construction contributed 68% to carcinogenic effect, 30% to the ozone depletion, 34% to the GWP, 35% to the non-carcinogenic effects, and 39% to the respiratory effects, respectively (Jeong et al., 2015). This discrepancy is caused by the fundamentally different systems and underlining data sets. Although the distribution network is longer and more material intensive in Cincinnati than in Atlanta, higher energy use in the Cincinnati system dominated most of the life cycle impact categories and over-shadowed the impacts of infrastructure. While we computed the LCI of infrastructure based on pipe materials, pipe lengths, treatment tanks' and storage units' dimensions, and pump types provided by the local utilities, the Atlanta study relied on European infrastructure datasets for pump stations, water and wastewater treatment plants, and pipes networks for distribution and collection (Jeong et al., 2015). Our assessment based on real utility data provides a more detailed LCI and stage contribution analyses (Table S18), which is valuable for improving the targeted process management without compromising the systematic view.

This study found that the GWP of wastewater infrastructure was less than 2% of the global warming impact caused by the wastewater subsystem, which lies at the lower end of the reported range of 1%–30% (Machado et al., 2007; Morera et al. 2016, 2017; Ortiz et al., 2007; Renou et al., 2008., Risch et al., 2015; Vlasopoulos et al., 2006). The wide range of infrastructure contribution partially reflects the inherent system variability (such as different treatment technologies and infrastructure characteristics), and distinct data sources. Various data sources including the existing ecoinvent database, equipment designs, manufacturer and supplier questionnaire responses, and real information from existing systems were utilized to estimate life cycle environmental impacts of infrastructure. This study, along with two previous studies (Morera et al. 2016, 2017), collected detailed information on construction materials, equipment, devices, and civil works for wastewater infrastructure. In addition, our study indicates that the contribution of water infrastructure to total GWP was 1.6 times higher than the contribution of wastewater infrastructure due to the extensive pipelines for water distribution in greater Cincinnati region.

### 3.3 Sensitivity analysis

**Fig. 4:**
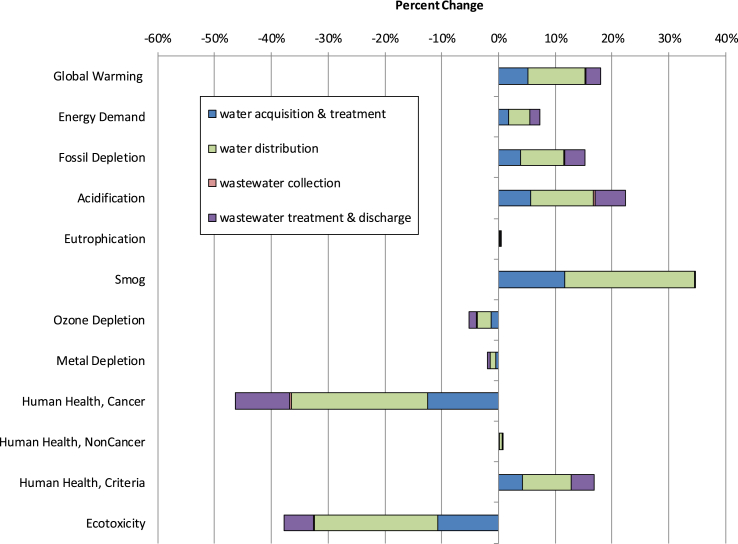
Percentage change of life cycle assessment impacts due to switching from Reliability First Corporation West electrical grid to U.S. average electrical grid.

Among the investigated factors in Table S22, the choice of electricity mix was the most influential factor to life cycle environmental impacts. The U.S. average and Reliability First Corporation West (RFCW) grids represent the national average and local conditions of electricity production, respectively. The U.S. average electricity mix was used in the baseline analysis such that results would be more applicable to facilities across the country. The changes of life cycle environmental impacts due to replacing the local RFCW grid with the U.S. average grid is shown in Fig. 4. While the switch from the RFCW electricity to U.S. average grid resulted in up to a 36% decline of the total smog impact due to the decreased usage of coal, this change had negligible impacts (less than 2%) on eutrophication, metal depletion, and human health noncancer impacts. In addition, this change of electricity mixes caused 7%–19% of decreases for global warming, energy demand, fossil depletion, acidification, and ozone depletion criteria impacts. The decreases of global warming, energy demand, fossil depletion, acidification, and ozone depletion were due to less use of coal in the U.S. average grid compared to the RFCW grid. However, the switch from RFCW electricity to national average increased human health cancer and ecotoxicity impacts. The U.S. average electricity contains a higher percentage of natural gas than RFCW electricity (Table S21), which caused the higher ecotoxicity and human health cancer impact compared to using the U.S. average electricity grid. The impact increases for the U.S. average electrical grid were specifically due to dioxin and aromatic hydrocarbon emissions associated with the extraction and production of natural gas.

**Fig. 5:**
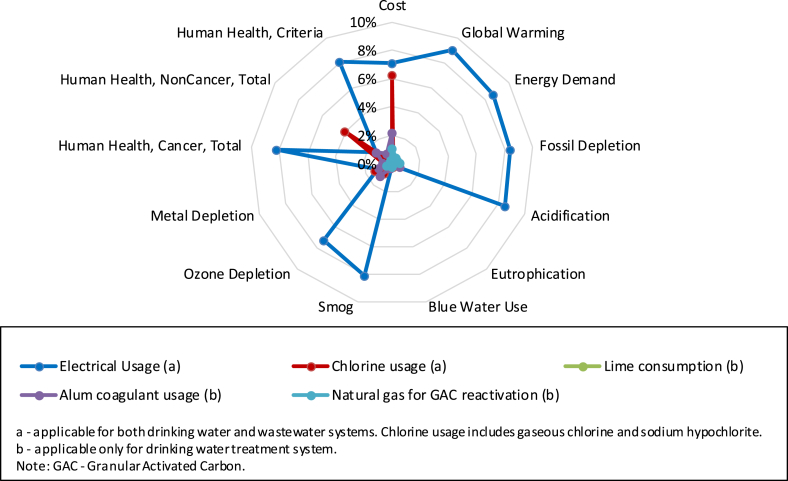
Percentage change for sensitivity analyses applicable to both life cycle assessment and life cycle cost results. All results except electricity are from minimum and maximum data collected from utilities. Electrical usage was varied ±10%.

Fig. 5 displays the sensitivity of the water system to various changes in electricity and chemical consumption. Following electricity mix, electricity consumption ranked as the second most influential input parameter. The electricity usage sensitivity assessed net impacts when overall purchased electricity consumption was varied by ±10%. As shown in Fig. S4, changing the total electricity used for water distribution changed the total impacts of entire water and wastewater systems up to ±4.5%. On the contrary, varying the total electricity used in wastewater by ±10% resulted in up to ±0.04% of the total GWP. The distinct sensitivity results for water distribution and wastewater treatment were due to the significant electricity usage in water distribution and wastewater treatment. By reducing the usage of electricity or switching to renewable energy, the total water treatment impacts could be greatly reduced. While the GWP of water distribution was influenced by electricity consumption to convey water, the GHG emitted from wastewater treatment was mainly related to the electricity and natural gas use during aeration and sludge incineration, and N_2_O emissions during sludge incineration. In addition, eutrophication and metal depletion were not sensitive to the electricity usage, as they were driven by wastewater effluent quality and infrastructure, respectively.

Compared to electricity mix and usage, chemical and material usage had negligible impacts on LCA findings. As shown in Fig. 5, varying material usages such as natural gas, chlorine, alum, lime, and sodium hypochlorite consumptions by minimum and maximum values reported by the utilities resulted in a less than ±2% change on environmental impacts for the majority of impact categories. Costs results, however, were sensitive to the amount of gaseous chlorine and sodium hypochlorite used in the drinking water and wastewater process. Human heath noncancer results were also moderately sensitive to the amount of sodium hypochlorite used for WWTP disinfection (±2.4%). Similarly, influences of infrastructure lifetime were minimal with the exception of metal depletion impact category (Table S24). The varied infrastructure lifetime resulted in a significant change of metal depletion impact, ranging from −10% to 17%. In addition, the cost of water and wastewater systems was sensitive to electricity unit cost and consumption. Electricity unit cost was the dominant parameter for total cost (Fig. S3). By varying electricity unit cost by ±20%, the total operational cost can change ±7% accordingly.

## 4 Discussion

**Fig. 6:**
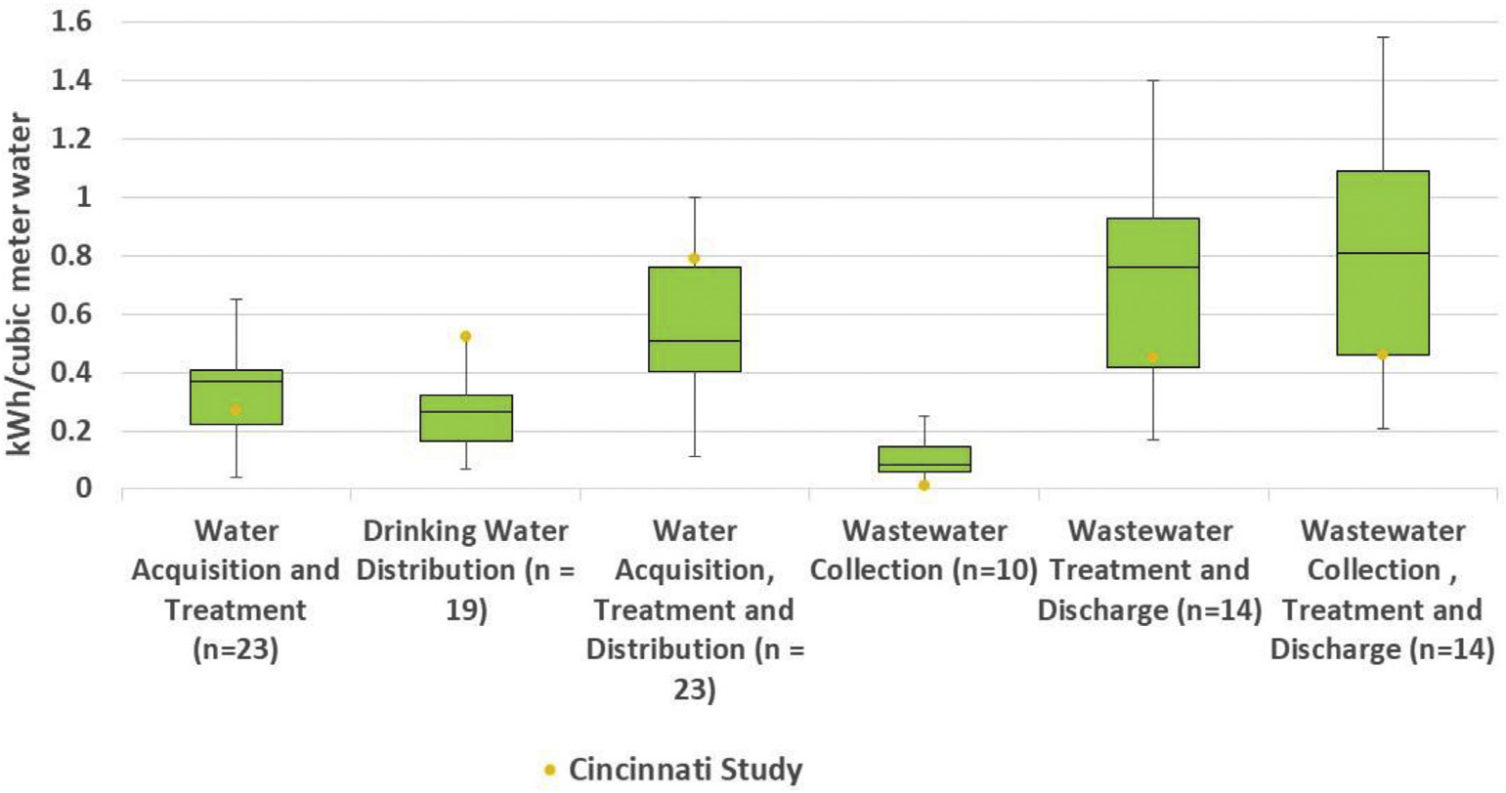
Electricity consumption of water systems in this study and existing literature.

Based upon our analysis and values currently reflected in the literature (Fig. 6), the energy consumption of the whole water system in North America presents great variability. A previous study focused on energy consumption GWP of entire water and wastewater systems in the U.S. and reported that the total direct electricity consumption spans from 0.32 to 1.43 kWh/m^3^ (Arpke and Hutzler, 2006). Our estimate of 1.25 kWh/m^3^ resides in the higher end of the reported range. Another study determined electricity intensity of 0.615 kWh/m^3^ is required for the entire water and wastewater system in Atlanta (Jeong et al., 2015), which is much lower than the average electricity consumption in Cincinnati systems. GCWW's Richard Miller plant in Cincinnati acquires most of its intake water from the Ohio River, which is a receiver of upstream municipal wastewater discharges, sanitary sewer overflows, and urban and agricultural storm water runoffs. In order to achieve high drinking water quality, energy-intensive GAC was employed. GCWW employed a UV disinfection process in 2013 to further ensure the water quality. Although UV disinfection is not included in this study, one would expect its addition will require more energy use and have more corresponding environmental impacts to produce the final treated drinking water.

The leading contributors to the life cycle fossil energy depletion of the whole urban water system were not consistent in previous studies. Our Cincinnati case study demonstrated that drinking water distribution was the top contributor to energy depletion, fossil depletion, acidification, smog, ozone depletion, metal depletion, human health cancer, and criteria pollutants categories (Fig. 2). Due to the massive piping network needed for transporting the treated water uphill over a large service area in the Greater Cincinnati area, the distribution system within the whole urban water system is the most energy-intensive stage. Electricity of 0.94 kWh/m^3^ was spent by Richard Miller water treatment plant to treat and distribute drinking water, which is much higher than the 0.11–0.66 kWh/m^3^ range (Arpke and Hutzler, 2006). Similarly, the energy consumption for water distribution in Cincinnati also exceeded the average value (0.38 kwh/m^3^) provided by the River Net Work report, which is based on multiple surveys across the U.S. (River Network, 2009).

Two previous studies (Friedrich et al., 2009), (Amores et al., 2013) agreed with our study in that the water distribution stage ranked as the largest contributor to multiple categories (energy demand, fossil depletion, acidification, smog, ozone depletion, metal depletion, human health cancer, and human health criteria pollutants). In contrast, other studies (Lassaux et al., 2007; Lundie et al., 2004, Mahgoub et al., 2010) identified the wastewater treatment and disposal stage as the largest contribution to energy consumption and had most of the environmental impacts. This discrepancy is caused by the differences in energy intensity of drinking water distribution stages in the different regions studied. Our study indicated a higher energy consumption of the drinking water distribution system in Cincinnati than other regions.

Despite the variation in energy use in drinking water treatment, the centralized treatment configuration is designed to achieve one water quality standard. It is inherently inefficient because of many lower quality non-potable use such as firefighting, periodical system flushing, irrigation, clothes washing and toilet flushing. Such centralized configuration also makes it unavoidable to convey water over long distance. As urban sprawl has been an inevitable phenomenon in city development, such long distance transporting water becomes extremely energy intensive. Alternatively, drinking water systems can be designed by incorporating concepts of decentralization and ‘fit for purpose’. For example, decentralized non-potable water reuse could not only improve system efficiency by reducing the degree of treatment required (which is important in a city with relatively poor quality of source water for non-potable purposes), but also eliminating the pipe network required to distribute large quantities of water.

The choice of distinct impact assessment methods may result in large discrepancies in eutrophication, human health and ecotoxicity categories (Hospido et al., 2012; Pizzol et al., 2011). In this study the TRACI 2.0 and ReCiPe 2008 methodology were employed, whereas a number of studies (Amores et al., 2013; Friedrich et al., 2009; Lassaux et al., 2007; Lundie et al., 2004, Mahgoub et al., 2010) used the Eco-indicator 99 (Goedkoop and Spriensma, 1999) or the CML (Guinee et al., 2001) methodologies. Within this study, characterization factors for human health and ecotoxicity categories were obtained from the TRACI model, which are the same as toxicity characterization factors in the USEtox model (US Environmental Protection Agency, 2014). Often, the USEtox model is considered a best practice methodology for ecotoxicity and human toxicity for LCIA (Corominas et al., 2013). Most LCAs for the entire water and wastewater systems have not reported life cycle water use inventory nor conducted water stress impact assessment due to absent datasets and large uncertainty of current impact assessment approaches. This comprehensive study focused on the life cycle blue water inventory and did not attempt to calculate water stress impact. Two studies (Amores et al., 2013) (Jeong et al., 2015), applied the water stress indicator proposed by previous studies (Canals et al., 2009; Pfister et al., 2009). This is a fast developing research area to create, apply and validate life cycle water use inventory and water stress impact assessment approaches (Núñez et al., 2016). The transparent inventory of blue water in this study fills in some data gaps for future development of inventory and impact assessment in water stress/scarcity and will give local decision makers relevant data to create better informed management solutions.

The whole urban water system results in diverse environmental impacts ranging from natural resources depletion to air and water quality degradation and associated negative human health impacts. Focusing on a single or a few impact categories such as energy consumption or GWP is insufficient to describe the holistic environmental impacts of water systems. In order to understand the diverse impacts of urban water systems and to avoid potential tradeoffs, it is necessary to perform LCA for a suite of impact categories. Moreover, the top contributing processes varied for different impact categories, which suggests that different processes should be targeted in order to minimize the corresponding environmental impacts. For example, minimizing electricity consumption will reduce fossil fuel depletion, acidification, smog, ozone depletion, human health cancer and criteria pollutants impacts. In contrast, reducing nutrients in wastewater effluents will mitigate the eutrophication impact. In addition, the comparison with previous studies indicate that environmental impacts of urban water systems are case-specific. In the Greater Cincinnati area, the extensive distribution piping network and the fact that drinking water is delivered uphill owing to the location of the plant at the bottom of the Ohio River valley result in high energy use in water conveyance. Energy saving efforts can be made to renew more efficient pumps, optimize the distribution network performance and reduce losses in the network or incorporate decentralization concepts in water supply (Cashman et al., 2018; Ma et al., 2015). The high impacts contributed from drinking water acquisition and treatment is in part due to the source water quality from Ohio river. Upstream source water protection will improve water quality and minimize treatment cost. Electricity use in wastewater treatment also contributes significantly to various environmental impacts. Strategies such as stormwater diversion, energy recovery through anaerobic digestion could reduce and offset the energy use in the plant and lower the end of life disposal cost. This case study shows when available, real datasets for infrastructure and operation & maintenance should be collected from utilities for a truly representative LCI and such targeted analyses provides bigger picture of urban water management as well as specific optimization potentials to improve overall system efficiency. Caution should be taken when generic databases are applied to assess the environmental impacts of a particular water system.

## 5 Conclusions

Based upon the detailed utility datasets, this study holistically quantified life cycle impacts and costs of urban water systems. Such comprehensive analyses provide insights for further LCA studies, and provide scientific basis to support utilities managers in effective and balanced decision making.

• This study emphasizes importance of collecting case-specific LCI for supporting effective decision making. The generic values from commercial LCA databases and average/median values of existing literature are incapable of accurately representing local conditions. Using average/median values of literature may result in misleading life cycle impacts and hampering effective decision making. For example, the energy use of drinking water distribution in Greater Cincinnati far exceeded the median value of the previous studies on urban drinking water systems. While the energy consumption of wastewater collection and treatment in Greater Cincinnati was much smaller than the median value of previous studies on urban wastewater systems. Based on the utility datasets, one may conclude that electricity for drinking water system should be prioritized for reduction within the urban water systems. However, if solely relying on the literature values, a contradictory suggestion that wastewater system should be prioritized for mitigating energy consumption may misguide decision making. In fact, the synthesis of existing studies indicates that energy consumption of urban water systems, vary significantly due to differences in treatment processes, geographical context, and management strategies. Case-specific datasets for infrastructure and operation & maintenance should be collected from utilities for truly representative life cycle inventory.
• In order to understand the diverse impacts of urban water systems and to provide scientific basis for the balanced decision support, it is necessary to perform LCA and LCC of entire water and wastewater systems for a suite of impact categories. Such comprehensive analyses pinpoint the relative contributions of treatment stages, and identify the top contributing processes for various impact categories. For example, electricity use is a key contributor to life cycle fossil fuel depletion, acidification, smog, ozone depletion, human health cancer and criteria pollutants impacts. In contrast, nutrient discharge in wastewater effluents dominates life cycle eutrophication impact.
• The analyses of stage contributions from various perspectives (such as unit processes, supply chain components, and life cycle stages) are critical to reveal top contributors for identifying targeted mitigation strategies. Based on relative contributions of unit processes, minimizing electricity consumption will reduce fossil fuel depletion, acidification, smog, ozone depletion, human health cancer and criteria pollutants impacts. Comparing the contributions of on-site and supply chain activities, we found that on-site activities significantly influence only blue water footprint and eutrophication impacts. The majority of life cycle environmental impacts were driven by the supply chain activities such as energy and chemical production, which are usually beyond the authority of water and wastewater utilities. Regarding the contributions of infrastructure and operational stages, this detailed analysis led to the conclusion that the contributions of infrastructure stage to environmental impacts were dwarfed by the operational stage, with the exception of metal depletion and human noncancer impact categories. Overall, reducing water withdrawal and improving discharge quality during operation stage can lower the local impacts of urban water services, while targeting energy-intensive unit process during operation stage can lower the supply chain impacts of urban water services.
• Sensitivity analyses demonstrated the cost of water and wastewater systems was sensitive to electricity mix, unit cost and consumption. This analysis provides insights for communities from other regions with different electricity mix and price. Energy saving throughout the system can greatly improve the system efficiency.
• Adopting cleaner energy sources, reducing electricity use, designing system configuration with fit-for-purpose, resource recovery and decentralization concepts are effective strategies to mitigate the environmental and economic impacts of urban water systems. This study and its framework provides a necessary benchmark to which future system improvements can be compared.

## Disclaimer

The views expressed in this article are those of the authors and do not necessarily represent the views or policies of the U.S. Environmental Protection Agency. Mention of trade names, products, or services does not convey, and should not be interpreted as conveying, official EPA approval, endorsement or recommendation.

## Declaration of interests

The authors declare that they have no known competing financial interests or personal relationships that could have appeared to influence the work reported in this paper.

The authors declare the following financial interests/personal relationships which may be considered as potential competing interests:

## Appendix A Supplementary data

APPLICATION APPLICATION
