# Supplementary material for: Holistic analysis of urban water systems in the Greater Cincinnati region: (1) life cycle assessment and cost implications
Source: Water Res X. 2018 Dec 14;2:100015. doi: 10.1016/j.wroa.2018.100015 (PMC6415537; doi:10.1016/j.wroa.2018.100015)
Supplement: APPLICATION [file mmc1.docx]

Supporting Information

Holistic Analysis of Urban Water Systems in the Greater Cincinnati Region: (1) Life Cycle Assessment and Cost Implications

Xiaobo Xue^a^, Sarah Cashman^b^, Anthony Gaglione^b^, Janet Mosley^b^, Lori Weiss^b^, Xin (Cissy) Ma^c,^*, Jennifer Cashdollar^d^, and Jay Garland^d^

^a^ Department of Environmental Health Sciences, School of Public Health, State University of New York at Albany, 1 University Drive, Rensselaer, NY, 12144

^b^ Eastern Research Group, Inc. (ERG), 110 Hartwell Avenue, Lexington, MA 02421

^c^ National Risk Management Research Laboratory, U.S. Environmental Protection Agency Office of Research and Development, 26 W Martin Luther King Drive, Cincinnati, OH 45268

^d^ National Exposure Research Laboratory, U.S. Environmental Protection Agency, Office of Research and Development, 26 W Martin Luther King Drive, Cincinnati, OH 45268

* Corresponding author, ma.cissy@epa.gov

The supporting information contains the system diagrams, influent and effluent quality of treatment plants, all relevant inputs and background calculations for infrastructure and operation stages of the water and wastewater systems. Detailed results are also provided.

Table of Contents:

1. Process diagrams for water and wastewater systems in Cincinnati
2. Influent and effluent quality of Richard Miller water treatment plant and Milk Creek wastewater treatment plant
3. Input and output flows for water processes during operation stage
4. Inputs for water infrastructure used in water acquisition, treatment and distribution
5. Input and output flows for wastewater processes during operation stage
6. Inputs for wastewater infrastructure used in wastewater collection, treatment and discharge
7. Assumptions of life time for infrastructure components
8. Life cycle impact categories and impact assessment tools
9. Life cycle assessment Results and contributions of unit processes
10. Contributions of infrastructure stage at unit process level
11. Top contributing unit process and pollutant for each impact category
12. Electricity mixes for US average and Cincinnati region
13. Sensitivity analysis results
14. Comparison with previous studies
15. Process diagrams for the water and wastewater treatment systems

The process diagrams describe the related water and wastewater treatment processes investigated in this study.

Figure S1. Unit processes of water extraction, treatment, and distribution stages

Figure S2. Unit processes of wastewater collection, treatment and discharge stages

Figure S1 and S2 describe the unit processes of water and wastewater systems in Cincinnati, Ohio for the year 2011. Once the raw water is pumped from the river, it proceeds with coagulation and settling stage with alum sulfate and sedimentation processes to eliminate suspended solids. The resulting sludge is successively thickened, centrifuged, and disposed to the Ohio River. The water treatment process continues through filtration which removes the remaining solids from water using a sand filter. Effluent from filtration goes through adsorption which adsorbs micro-pollutants (e.g. pesticides and residual organic matter) by a granular activated carbon system. Further conditioning of water is performed by adding sodium hydroxide and sodium hexametaphosphate to adjust pH. The following step of disinfection with chlorine is designed to kill microorganisms. Finally, the last treatment stage before water distribution consists of a fluoridation stage with the addition of sodium fluorosilicate. Sodium hypochlorite is added at certain points in the distribution system to boost chlorine levels. After water is used at households, sewage and storm runoff are collected through a combined centralized system. Combined sewer treatment involves three major treatment stages. During pretreatment stage, the influent in sewage water passes through bar screens to remove large objects and then through grit channels to eliminate the sand and grit. As a following step, sewage flows through primary sedimentation tanks which are used to settle sludge. The sludge is thickened and dewatered, and then incinerated into ash which is disposed in a landfill. Thereafter, the settled sewage liquor flows to the aeration tanks where microorganisms convert the biodegradable soluble organic contaminants into carbon dioxide. The less soluble fractions bond into floc and settle in the secondary clarifiers. The last step prior to the wastewater discharge includes disinfection with sodium hypochlorite.

1. **Influent and effluent quality of Richard Miller water treatment plant and Milk Creek wastewater treatment plant**

Table S1. Incoming and outgoing water quality metrics for GCWW Richard Miller Treatment Plant (per m^3^water)

|  | Incoming Water | | | Outgoing Water | | |  |
| --- | --- | --- | --- | --- | --- | --- | --- |
| *Water Metrics* | Minimum | Maximum | Average | Minimum | Maximum | Average | Unit (per m^3^ water) |
| Ammonia | <0.010 | 0.19 | 0.050 | 0 | 0 | 0 | g |
| Arsenic | <0.001 | 0.0016 | <0.001 | <0.001 | <0.001 | <0.001 | g |
| Chromium | <5.0E-04 | 0.0029 | 0.0010 | <0.010 | <0.010 | <0.010 | g |
| Dissolved organic carbon | 2.50 | 3.30 | 2.99 | 0.61 | 1.01 | 0.94 | g |
| Dissolved solids | 158 | 299 | 229 | 132 | 317 | 228 | g |
| Iron | 0.30 | 0.30 | 0.30 | <0.020 | <0.020 | <0.020 | g |
| Manganese | 0.053 | 0.053 | 0.053 | <0.010 | <0.010 | <0.010 | g |
| Nitrate | 0.63 | 1.14 | 0.89 | 0.62 | 1.06 | 0.86 | g |
| pH | 7.50 | 8.40 | 7.80 | 8.20 | 8.80 | 8.80 | pH |
| Phosphorus | 0.030 | 0.11 | 0.060 | 0.15 | 0.20 | 0.17 | g |
| Suspended solids | 1.90 | 225 | 43.1 | 0 | 0 | 0 | g |
| Temperature | 5.10 | 33.0 | 18.0 | 4.70 | 29.0 | 17.0 | ºC |
| Total organic carbon | 2.10 | 4.80 | 3.05 | 0.40 | 1.43 | 0.85 | g |
| Turbidity | 2.40 | 307 | 46.0 | 0.050 | 0.13 | 0.070 | NTU |
| TTHM | <5.0E-04 | <5.0E-04 | <5.0E-04 | 0.0078 | 0.020 | 0.016 | g |
| Chlorine | 0 | 0 | 0 | 1.13 | 1.66 | 1.37 | g |
| Cryptosporidium | <20.0 | <91.0 | <51.0 | <0.80 | <1.10 | <1.00 | oocysts |
| Giardia | <20.0 | 200 | 20.0 | <0.80 | <1.10 | <1.00 | oocysts |
| E. coli | 0 | 6,030,000 | 1,340,000 | 0 | 0 | 0 | counts |
| Heterotrophic plate count | 0 | 14,000,000,000 | 2,250,000,000 | 0 | 0 | 0 | counts |
| *Source: GCWW primary data collection for the year 2011.*    Table S2. Incoming and outgoing water quality metrics for MSDGC Mill Creek Plant (per m^3^ water)   \|  \| Incoming Water \| \| \| Outgoing Water \| \| \|  \| \| --- \| --- \| --- \| --- \| --- \| --- \| --- \| --- \| \| *Water Metrics* \| Minimum \| Maximum \| Average \| Minimum \| Maximum \| Average \| Unit \| \| Ammonia \|  \|  \|  \| 2.20 \| 11.4 \| 7.66 \| g \| \| Dissolved solids \|  \|  \|  \| 4.90 \| 5.80 \| 5.20 \| g \| \| pH \| 5.90 \| 7.60 \| 6.80 \|  \|  \|  \| pH \| \| Phosphorus \|  \|  \|  \| 0.22 \| 0.88 \| 0.55 \| g \| \| Suspended solids \| 46.0 \| 1,072 \| 208 \| 13.0 \| 32.0 \| 21.5 \| g \| \| Temperature \| 8.00 \| 23.0 \| 16.0 \| 13.4 \| 23.8 \| 18.0 \| ºC \| \| Turbidity \|  \|  \|  \| 3.40 \| 78.0 \| 9.20 \| NTU \| \| *Source: Primary data collected from MSDGC for the year 2011* \| \| \| \| \| \| \| \| | | | | | | | |

1. **Input and output flows for water treatment processes during operation stage**

The energy, material, and transportation inputs for operation stages of water treatment and distribution processes are documented in Table S3.

Table S3. Drinking water treatment life cycle inventory model-input and output operational data (per m^3^ drinking water delivered to consumer)

|  |  | **Unit** | **TOTAL** | **Quantity by Life Cycle Stage** | | | | | | | | | | | | | | |
| --- | --- | --- | --- | --- | --- | --- | --- | --- | --- | --- | --- | --- | --- | --- | --- | --- | --- | --- |
|  |  |  | **Quantity** | **Source Water Acquisition** | **Energy for Pumping** | **Flocculation** | **Sedimentation** | **Disposal, Sedimentation Waste** | **Lime Addition** | **Filtration** | **Adsorption** | **GAC Reactivation** | **Conditioning** | **Primary Disinfection, Gaseous Chlorine** | **Fluoridation** | **Distribution** | **Transport, Treated Drinking Water, Water Supply Pipeline** | **Drinking Water Consumption** |
| **Inputs** | | | | | | | | | | | | | | | | | | |
| Primary Raw Material | Raw water, river | m^3^ | **1.19** | 1.19 |  |  |  |  |  |  |  |  |  |  |  |  |  |  |
| Energy | Purchased electricity | kWh | **0.94** |  | 0.32 |  |  |  |  |  |  |  |  |  |  |  | 0.62 |  |
|  | Electricity from on-site hydroelectric cogeneration | kWh | **0.019** |  | 0.019 |  |  |  |  |  |  |  |  |  |  |  |  |  |
|  | Natural gas | m^3^ | **0.0026** |  |  |  |  |  |  |  |  | 0.0026 |  |  |  |  |  |  |
| Material and Chemical Inputs | Alum coagulant, 48% aluminum sulfate | kg | **0.019** |  |  | 0.019 |  |  |  |  |  |  |  |  |  |  |  |  |
|  | Polymer (polyDADMAC, 10%) | kg | **0.0021** |  |  | 0.0021 |  |  |  |  |  |  |  |  |  |  |  |  |
|  | Ferric sulfate | kg | **0.0014** |  |  |  | 0.0014 |  |  |  |  |  |  |  |  |  |  |  |
|  | Quicklime at plant | kg | **0.0032** |  |  |  |  |  | 0.0032 |  |  |  |  |  |  |  |  |  |
|  | Sand | kg | **0.0082** |  |  |  |  |  |  | 0.0082 |  |  |  |  |  |  |  |  |
|  | GAC from bituminous coal | kg | **0.0030** |  |  |  |  |  |  |  | 0.0030 |  |  |  |  |  |  |  |
|  | Sodium hypochlorite, 15% | kg | **5.0E-04** |  |  |  |  |  |  |  |  |  |  |  |  | 5.0E-04 |  |  |
|  | Sodium hydroxide, 50% | kg | **0.027** |  |  |  |  |  |  |  |  |  | 0.027 |  |  |  |  |  |
|  | Sodium hexametaphosphate, 30% | kg | **0.0024** |  |  |  |  |  |  |  |  |  | 0.0024 |  |  |  |  |  |
|  | Gaseous chlorine | kg | **0.0021** |  |  |  |  |  |  |  |  |  |  | 0.0021 |  |  |  |  |
|  | Hydrofluorosilicic acid, 24% | kg | **0.0051** |  |  |  |  |  |  |  |  |  |  |  | 0.0051 |  |  |  |
| Transport | Combination truck transport, alum coagulant | tkm | **0.0012** |  |  | 0.0012 |  |  |  |  |  |  |  |  |  |  |  |  |
|  | Combination truck transport, lime | tkm | **1.2E-04** |  |  |  |  |  | 1.2E-04 |  |  |  |  |  |  |  |  |  |
|  | Combination truck transport, ferric sulfate | tkm | **8.6E-04** |  |  |  | 8.6E-04 |  |  |  |  |  |  |  |  |  |  |  |
|  | Combination truck transport, gaseous chlorine | tkm | **1.3E-04** |  |  |  |  |  |  |  |  |  |  | 1.3E-04 |  |  |  |  |
|  | Rail transport, gaseous chlorine | tkm | **7.3E-04** |  |  |  |  |  |  |  |  |  |  | 7.3E-04 |  |  |  |  |
|  | Combination truck transport, hydrofluorosilicic acid | tkm | **1.2E-04** |  |  |  |  |  |  |  |  |  |  |  | 1.2E-04 |  |  |  |
|  | Rail transport, hydrofluorosilicic acid | tkm | **0.0076** |  |  |  |  |  |  |  |  |  |  |  | 0.0076 |  |  |  |
|  | Combination truck transport, sodium hypochlorite | tkm | **2.7E-05** |  |  |  |  |  |  |  |  |  |  |  |  | 2.7E-05 |  |  |
|  | Combination truck transport, sodium hydroxide | tkm | **2.7E-05** |  |  |  |  |  |  |  |  |  | 2.7E-05 |  |  |  |  |  |
|  | Barge transport, sodium hydroxide | tkm | **6.0E-04** |  |  |  |  |  |  |  |  |  | 6.0E-04 |  |  |  |  |  |
|  | Combination truck transport sodium hexametaphosphate | tkm | **1.3E-04** |  |  |  |  |  |  |  |  |  | 1.3E-04 |  |  |  |  |  |
|  | Combination truck transport polymer (polyDADMAC) | tkm | **9.8E-04** |  |  | 9.8E-04 |  |  |  |  |  |  |  |  |  |  |  |  |
|  | Combination truck transport GAC | tkm | **6.5E-04** |  |  |  |  |  |  |  | 6.5E-04 |  |  |  |  |  |  |  |
|  | Water supply pipeline transport | tkm | **6,027** |  |  |  |  |  |  |  |  |  |  |  |  | 6,027 |  |  |
| **Outputs** | | | | | | | | | | | | | | | | | | |
| Waste & Loss | Disposal of sedimentation waste | liters | **0.048** |  |  |  | 0.048 |  |  |  |  |  |  |  |  |  |  |  |
|  | Water loss | m^3^ | **0.19** | 0.0036 |  |  |  |  |  |  |  |  |  |  |  | 0.19 |  |  |
| Water Emissions | Aluminum (water emissions) | kg | **0.0016** |  |  |  |  | 0.0016 |  |  |  |  |  |  |  |  |  |  |
|  | Ammonia (water emission) | kg | **3.6E-06** |  |  |  |  | 3.6E-06 |  |  |  |  |  |  |  |  |  |  |
|  | Biological oxygen demand (water emission) | kg | **3.9E-04** |  |  |  |  | 3.9E-04 |  |  |  |  |  |  |  |  |  |  |
|  | Chemical oxygen demand (water emission) | kg | **0.0081** |  |  |  |  | 0.0081 |  |  |  |  |  |  |  |  |  |  |
|  | Suspended solids (water emission) | kg | **0.016** |  |  |  |  | 0.016 |  |  |  |  |  |  |  |  |  |  |
| Air Emissions | Carbon monoxide (air emission) | kg | **2.7E-05** |  |  |  |  |  |  |  |  | 2.7E-05 |  |  |  |  |  |  |
|  | Nitrogen oxides (air emission) | kg | **9.2E-05** |  |  |  |  |  |  |  |  | 9.2E-05 |  |  |  |  |  |  |
|  | Particulates, <10 um (air emission) | kg | **1.5E-05** |  |  |  |  |  |  |  |  | 1.5E-05 |  |  |  |  |  |  |
|  | Particulates, <2.5 um (air emission) | kg | **1.5E-05** |  |  |  |  |  |  |  |  | 1.5E-05 |  |  |  |  |  |  |
|  | Sulfur oxides (air emission) | kg | **2.9E-04** |  |  |  |  |  |  |  |  | 2.9E-04 |  |  |  |  |  |  |
|  | Volatile organic compounds (air emission) | kg | **1.3E-05** |  |  |  |  |  |  |  |  | 1.3E-05 |  |  |  |  |  |  |
| Final Product | Drinking water delivered to consumer | m^3^ | **1.00** |  |  |  |  |  |  |  |  |  |  |  |  |  |  | 1.00 |

*Source: GCWW primary data collection from the year 2011*.

1. **Inputs for water infrastructure used in water acquisition, treatment and distribution**

The Tables S4-S7 describe the material inputs at the unit process level for structures at the drinking water treatment facility, on-site piping for water acquisition and treatment, and water distribution systems including pipes, storage, motors, pumps, and valves.

Table S4. Infrastructure requirements for drinking water treatment plant buildings and features (per m^3^ water delivered to consumer)

|  |  | **Material Type** | | | | | | |
| --- | --- | --- | --- | --- | --- | --- | --- | --- |
| **Life Cycle Stage** | **Infrastructure Component** | *Earthworks (m^3^)* | *Reinforcing Steel (kg)* | *6.5' Concrete piping (m)* | *Concrete (m^3^)* | *Bricks (kg)* | *Limestone (kg)* |  |
| Source Water Acquisition | Intake 1 (to Pump Station 1) | 1.2E-05 | 0 | 0 | 0 | 9.4E-04 | 0.0012 |  |
|  | Intake 2 (to Pump Station 2) | 1.2E-05 | 0 | 0 | 0 | 9.4E-04 | 0.0012 |  |
|  | Pump Station 1 (Near River) | 8.9E-07 | 0 | 0 | 0 | 0 | 0.0024 |  |
|  | Pump Station 2 (Farther from River) | 3.3E-06 | 5.1E-05 | 6.9E-08 | 6.0E-07 | 0 | 0 |  |
| Flocculation | Pretreatment Complex | 7.4E-06 | 1.1E-04 | 1.5E-07 | 1.3E-06 | 0 | 0 |  |
| Sedimentation | Settling Reservoir #1 (Closer to Pump Station) | 4.8E-05 | 0 | 0 | 0 | 1.8E-04 | 0 |  |
|  | Settling Reservoir #2 (Farther from Pump Station) | 5.3E-05 | 0 | 0 | 0 | 1.9E-04 | 0 |  |
|  | Chemical House (East) | 9.3E-07 | 1.4E-05 | 1.9E-08 | 1.7E-07 | 0 | 0 |  |
|  | Clarification Basins | 6.4E-06 | 0 | 0 | 4.1E-07 | 0 | 0 |  |
| Filtration | Filter Building | 5.2E-06 | 8.0E-05 | 1.1E-07 | 9.3E-07 | 0 | 0 |  |
| Adsorption | GAC Facility | 1.4E-05 | 2.1E-04 | 2.9E-07 | 2.5E-06 | 0 | 0 |  |
| Conditioning | Caustic Soda Facility | 4.1E-06 | 6.4E-05 | 8.6E-08 | 7.4E-07 | 0 | 0 |  |
| Primary Disinfection | Chlorine Injector Facility | 2.6E-07 | 4.1E-06 | 5.5E-09 | 4.8E-08 | 0 | 0 |  |
| Fluoridation | Clearwell #1 | 9.9E-06 | 0 | 0 | 4.7E-07 | 0 | 0 |  |
|  | Clearwell #2 | 2.4E-06 | 0 | 0 | 1.8E-07 | 0 | 0 |  |

*Source: GCWW primary data collection with estimations made with facility map.*

Table S5. Infrastructure requirements for drinking water treatment plant on-site piping (per m^3^ water delivered to consumer)

|  |  | **Pipe Type** | | |  |  |
| --- | --- | --- | --- | --- | --- | --- |
| **Life Cycle Stage** | **Diameter** | *Gray Iron Pipe (m)* | *Ductile Iron Pipe (m)* | *Concrete Pipe (m)* | *Total Length (m)* | *Earthworks (m^3^)* |
| Source Water Acquisition | 7' | 0 | 0 | 9.8E-08 | 9.8E-08 | 1.0E-06 |
|  | 36" | 2.7E-09 | 1.6E-09 | 2.4E-10 | 4.6E-09 | 1.8E-08 |
|  | 50" | 3.8E-08 | 2.3E-08 | 3.4E-09 | 6.4E-08 | 3.5E-07 |
|  | 54" | 1.9E-09 | 1.2E-09 | 1.7E-10 | 3.3E-09 | 2.0E-08 |
|  | 72" | 1.2E-09 | 7.0E-10 | 1.0E-10 | 2.0E-09 | 1.7E-08 |
| Flocculation | 60" | 6.5E-09 | 4.0E-09 | 5.9E-10 | 1.1E-08 | 7.6E-08 |
|  | 72" | 7.4E-08 | 4.5E-08 | 6.7E-09 | 1.3E-07 | 1.1E-06 |
| Sedimentation | 36" | 2.3E-09 | 1.4E-09 | 2.1E-10 | 3.9E-09 | 1.5E-08 |
|  | 54" | 2.7E-09 | 1.6E-09 | 2.4E-10 | 4.6E-09 | 2.8E-08 |
|  | 60" | 7.3E-08 | 4.4E-08 | 6.6E-09 | 1.2E-07 | 8.4E-07 |
|  | 72" | 1.8E-08 | 1.1E-08 | 1.7E-09 | 3.1E-08 | 2.7E-07 |
|  | 60" | 3.7E-08 | 2.2E-08 | 3.3E-09 | 6.3E-08 | 4.3E-07 |
| Filtration | 36" | 1.2E-08 | 7.4E-09 | 1.1E-09 | 2.1E-08 | 7.0E-08 |
| Adsorption | 36" | 6.9E-09 | 4.2E-09 | 6.2E-10 | 1.2E-08 | 4.6E-08 |
| Conditioning | 36" | 3.1E-09 | 1.9E-09 | 2.8E-10 | 5.2E-09 | 2.1E-08 |
| Fluoridation | 36" | 1.5E-08 | 9.3E-09 | 1.4E-09 | 2.6E-08 | 1.0E-07 |
| *Source: GCWW primary data collection with estimations made with facility map.* | | | | | | |

Table S6. Infrastructure requirements for drinking water treatment distribution system piping (per m^3^ water delivered to consumer)

|  |  | **Pipe Type** | | | | | | | |  |  |
| --- | --- | --- | --- | --- | --- | --- | --- | --- | --- | --- | --- |
| **Life Cycle Stage** | **Diameter** | *Gray Iron (m)* | *Ductile Iron (m)* | *Concrete (m)* | *Steel (m)* | *Copper (m)* | *PVC (m)* | *HDPE (m)* | *Transite (m)* | *Total Length (m)* | *Earthworks (m^3^)* |
| Distribution | 0.75" | 2.30E-09 | 1.39E-09 | 2.07E-10 | 1.81E-11 | 4.78E-12 | 1.26E-12 | 5.41E-12 | 1.27E-11 | 3.95E-09 | 4.21E-09 |
|  | 1" | 6.76E-09 | 4.10E-09 | 6.09E-10 | 5.32E-11 | 1.41E-11 | 3.70E-12 | 1.59E-11 | 3.73E-11 | 1.16E-08 | 1.25E-08 |
|  | 1.5" | 1.06E-08 | 6.41E-09 | 9.52E-10 | 8.33E-11 | 2.20E-11 | 5.78E-12 | 2.49E-11 | 5.84E-11 | 1.81E-08 | 2.01E-08 |
|  | 2" | 2.90E-07 | 1.76E-07 | 2.61E-08 | 2.28E-09 | 6.03E-10 | 1.59E-10 | 6.82E-10 | 1.60E-09 | 4.97E-07 | 5.67E-07 |
|  | 2.5" | 0.00E+00 | 0.00E+00 | 0.00E+00 | 0.00E+00 | 0.00E+00 | 0.00E+00 | 0.00E+00 | 0.00E+00 | 0.00E+00 | 0.00E+00 |
|  | 3" | 1.88E-08 | 1.14E-08 | 1.69E-09 | 1.48E-10 | 3.90E-11 | 1.03E-11 | 4.41E-11 | 1.04E-10 | 3.22E-08 | 3.87E-08 |
|  | 4" | 1.52E-06 | 9.19E-07 | 1.37E-07 | 1.19E-08 | 3.15E-09 | 8.30E-10 | 3.57E-09 | 8.38E-09 | 2.60E-06 | 3.29E-06 |
|  | 6" | 7.52E-05 | 4.55E-05 | 6.77E-06 | 5.92E-07 | 1.56E-07 | 4.11E-08 | 1.77E-07 | 4.15E-07 | 1.29E-04 | 1.80E-04 |
|  | 8" | 9.28E-05 | 5.62E-05 | 8.36E-06 | 7.31E-07 | 1.93E-07 | 5.07E-08 | 2.18E-07 | 5.12E-07 | 1.59E-04 | 2.43E-04 |
|  | 10" | 4.05E-06 | 2.45E-06 | 3.65E-07 | 3.19E-08 | 8.41E-09 | 2.21E-09 | 9.52E-09 | 2.24E-08 | 6.94E-06 | 1.16E-05 |
|  | 12" | 3.21E-05 | 1.94E-05 | 2.89E-06 | 2.53E-07 | 6.67E-08 | 1.76E-08 | 7.55E-08 | 1.77E-07 | 5.50E-05 | 9.97E-05 |
|  | 16" | 3.85E-06 | 2.33E-06 | 3.47E-07 | 3.03E-08 | 8.00E-09 | 2.11E-09 | 9.06E-09 | 2.13E-08 | 6.60E-06 | 1.40E-05 |
|  | 20" | 8.10E-06 | 4.91E-06 | 7.29E-07 | 6.38E-08 | 1.68E-08 | 4.43E-09 | 1.90E-08 | 4.47E-08 | 1.39E-05 | 3.39E-05 |
|  | 24" | 4.71E-06 | 2.85E-06 | 4.24E-07 | 3.71E-08 | 9.78E-09 | 2.57E-09 | 1.11E-08 | 2.60E-08 | 8.07E-06 | 2.25E-05 |
|  | 30" | 9.96E-07 | 6.03E-07 | 8.97E-08 | 7.84E-09 | 2.07E-09 | 5.45E-10 | 2.34E-09 | 5.50E-09 | 1.71E-06 | 5.71E-06 |
|  | 35" | 3.98E-06 | 2.41E-06 | 3.58E-07 | 3.13E-08 | 8.26E-09 | 2.17E-09 | 9.35E-09 | 2.20E-08 | 6.82E-06 | 2.62E-05 |
|  | 36" | 4.55E-06 | 2.76E-06 | 4.10E-07 | 3.58E-08 | 9.45E-09 | 2.49E-09 | 1.07E-08 | 2.51E-08 | 7.80E-06 | 3.08E-05 |
|  | 42" | 9.26E-07 | 5.61E-07 | 8.34E-08 | 7.29E-09 | 1.92E-09 | 5.06E-10 | 2.18E-09 | 5.11E-09 | 1.59E-06 | 7.30E-06 |
|  | 44" | 2.95E-06 | 1.79E-06 | 2.66E-07 | 2.32E-08 | 6.13E-09 | 1.61E-09 | 6.94E-09 | 1.63E-08 | 5.06E-06 | 2.44E-05 |
|  | 46" | 3.57E-07 | 2.16E-07 | 3.22E-08 | 2.81E-09 | 7.42E-10 | 1.95E-10 | 8.40E-10 | 1.97E-09 | 6.13E-07 | 3.10E-06 |
|  | 48" | 1.29E-06 | 7.82E-07 | 1.16E-07 | 1.02E-08 | 2.68E-09 | 7.06E-10 | 3.04E-09 | 7.13E-09 | 2.21E-06 | 1.17E-05 |
|  | 54" | 2.73E-07 | 1.66E-07 | 2.46E-08 | 2.15E-09 | 5.68E-10 | 1.49E-10 | 6.42E-10 | 1.51E-09 | 4.68E-07 | 2.83E-06 |
|  | 60" | 3.06E-07 | 1.85E-07 | 2.76E-08 | 2.41E-09 | 6.36E-10 | 1.67E-10 | 7.19E-10 | 1.69E-09 | 5.25E-07 | 3.58E-06 |
| *Source: GCWW Primary data collection from 2011 water main inventory*. | | | | | | | | | | | |

Table S7. Infrastructure requirements for drinking water treatment distribution system water storage, motors, pumps, and valves (per m^3^ water delivered to consumer)

|  |  | **Material Type** | | | | | | | |
| --- | --- | --- | --- | --- | --- | --- | --- | --- | --- |
| **Life Cycle Stage** | **Infrastructure** | *Concrete (m^3^)* | *Steel (kg)* | *Earthworks (m^3^)* | *Electrical steel (kg)* | *Stainless 18/8 coil (kg)* | *Cast Iron (kg)* | *Aluminum (kg)* | *Copper (kg)* |
| Distribution | Water Storage Tanks | 3.2E-08 | 6.4E-05 | 0 | 0 | 0 | 0 | 0 | 0 |
|  | Reservoirs | 0 | 0 | 2.9E-05 | 0 | 0 | 0 | 0 | 0 |
|  | Motors | 0 | 8.8E-06 | 0 | 4.1E-05 | 0 | 3.9E-05 | 2.4E-06 | 7.1E-06 |
|  | Pumps | 0 | 0 | 0 | 0 | 4.5E-06 | 6.0E-05 | 0 | 0 |
|  | Valves | 0 | 0.0021 | 0 | 0 | 0 | 0 | 0 | 0 |
| *Source: GCWW primary data collection.* | | | | | | | | | |

1. Input and output flows for wastewater processes during operation stage

The energy, material, and transportation inputs for operation stages of wastewater collection and treatment processes are documented in Table S8. As shown in Table S8, we also estimated impacts from greenhouse gases (GHG) generated at the treatment plant. The Mill Creek Plant does not perform nutrient removal processes or anaerobic digestion, and sludge flows continuously through the sludge thickening processes to the incinerators. Therefore, we expect minimal contribution to methane and nitrous oxide emissions from the aeration and sludge thickening processes^[[1]](#footnote-1),^^[[2]](#footnote-2)^. We calculated both biogenic and non-biogenic CO_2_ emissions from aeration and all biogenic and fossil GHG emissions from the incineration process, but only used fossil GHG emission in the model. We estimated biogenic CO_2_ emissions from aeration using the method proposed by Monteith et al.^[[3]](#footnote-3)^ MSDGC provided information on volume of aerobic reactor volume, annual volume of influent wastewater, influent and effluent total suspended solids, and solids retention time while the paper from Monteith et al. supplied the remaining parameters of a typical conventional activated sludge treatment system needed for the calculation.

EPA used the following information to estimate GHG emissions from incineration for the base case:

- For biogenic CO_2_ emissions from sludge:
  - The Intergovernmental Panel on Climate Change (IPCC) 2006 Guidelines for GHG Inventories gives a range of 40 to 50% carbon content in dry sludge.^[[4]](#footnote-4)^ EPA used the average of this range (45%) in calculations.
  - The IPCC provides a default oxidation rate of 100%.17
  - The biogenic CO_2_ emissions factor was calculated as 1.65 tons biogenic CO_2_ / dry ton of sludge.
  - According to MSDGC, the Mill Creek Plant produces 37,811 metric tons of dry sludge and treats 157,615,342 m^3^ of wastewater annually.
  - EPA calculated that 0.40 kg biogenic CO_2_ is released per cubic meter of wastewater treated.
- For CH_4_ emissions from sludge:
  - The IPCC 2006 gives a default value of 4.85$\times$10^-5^ kg CH_4_ emitted/kg of dry sludge burned, which converts to 12 g CH_4_/m^3^ of wastewater treated.14^,^15^,^17
- For N_2_O emissions from sludge:
  - The Suzuki model describes nitrous oxide emissions from continuously operated fluidized bed incinerators using the equation: *η* = 161.3 - 0.140*T_f_*, where *η* is the percent of total N in the sludge that is volatilized as N_2_O, and *T_f_* is the average highest freeboard temperature from the fluidized bed facilities.
  - Based on the average highest freeboard temperature of 1,600 degrees F provided by Mill Creek Plant, *η* = 0.011034 and emissions of N_2_O are 6.936$\times$10^-4^ tons per dry ton of sludge incinerated.
  - The BEAM model uses a default ratio of 0.04 tons nitrogen per ton of dry sludge.^[[5]](#footnote-5)^
  - Total nitrous oxide emissions were calculated as 0.17 g N_2_O per cubic meter of wastewater treated at the plant.
- For fossil GHG emissions from natural gas combustion:
  - Emissions from natural gas combusted in Mill Creek’s incinerator are based on LCI data from the National Renewable Energy Laboratory’s U.S. Life Cycle Inventory Database (U.S. LCI), a publicly available life cycle inventory source.^[[6]](#footnote-6)^

Table S8. Wastewater treatment life cycle inventory model input and output operational data (per m^3^ wastewater treated)

| **Input** | **Unit** | **TOTAL**  **Quantity** | **Quantity by Life Cycle Stage** | | | | | | | | | | |
| --- | --- | --- | --- | --- | --- | --- | --- | --- | --- | --- | --- | --- | --- |
|  |  |  | **Wastewater Collection** | **Pumping at WWT Plant** | **Screening and Grit Removal** | **Primary Sedimentation** | **Sludge Thickening and Dewatering** | **Sludge Incineration** | **Aeration** | **Secondary Clarifiers** | **Disinfection** | **Mobile Fuel Combustion, at Plant** | **Release of Wastewater Effluent** |
|  |  |  |  |  |  |  |  |  |  |  |  |  |  |
| Industrial and household wastewater | m^3^ | **1.00** | 1.00 |  |  |  |  |  |  |  |  |  |  |
| Purchased electricity | kWh | **0.45** | 0.007 | 0.078 | 6.2E-04 | 0.0086 | 0.060 | 0.0066 | 0.28 | 0.0097 |  |  |  |
| Natural gas | m3 | **0.023** | 3.4E-04 |  |  |  |  | 0.023 |  |  |  |  |  |
| Diesel | liters | **0.0018** | 7.8E-04 |  |  |  |  |  |  |  |  | 0.001 |  |
| Gasoline | liters | **0.0015** | 0.0012 |  |  |  |  |  |  |  |  | 3.1E-04 |  |
| Sodium hypochlorite | liters | **0.012** |  |  |  |  |  |  |  |  | 0.012 |  |  |
| Sodium hydroxide | kg | **0.0020** |  |  |  | 0.0020 |  |  |  |  |  |  |  |
| Polymer (polyacrylamide) | kg | **0.0069** |  |  |  |  | 0.0069 |  |  |  |  |  |  |
| **Output** |  |  |  |  |  |  |  |  |  |  |  |  |  |
| Sludge cake (landfill waste disposal) | kg | **0.0045** |  |  |  |  | 0.0045 |  |  |  |  |  |  |
| Screenings, grit (landfill waste disposal) | kg | **0.029** |  |  | 0.029 |  |  |  |  |  |  |  |  |
| Ash (landfill waste disposal) | kg | **0.054** |  |  |  |  |  | 0.054 |  |  |  |  |  |
| Carbon monoxide (air emission) | kg | **5.9E-06** |  |  |  |  |  | 5.9E-06 |  |  |  |  |  |
| VOC (air emission) | kg | **2.8E-07** |  |  |  |  |  | 2.8E-07 |  |  |  |  |  |
| PM_2.5_ (air emission) | kg | **3.6E-06** |  |  |  |  |  | 3.6E-06 |  |  |  |  |  |
| PM_10_ (air emission) | kg | **4.2E-06** |  |  |  |  |  | 4.2E-06 |  |  |  |  |  |
| Lead (air emissions) | kg | **1.8E-09** |  |  |  |  |  | 1.8E-09 |  |  |  |  |  |
| Organic compounds (air emission) | kg | **3.1E-06** |  |  |  |  |  | 3.1E-06 |  |  |  |  |  |
| NOx (air emission) | kg | **8.9E-06** |  |  |  |  |  | 8.9E-06 |  |  |  |  |  |
| SO_2_ (air emission) | kg | **1.1E-06** |  |  |  |  |  | 1.1E-06 |  |  |  |  |  |
| Biogenic carbon dioxide (air emission) | kg | **0.50** |  |  |  |  |  | 0.40 | 0.099 |  |  |  |  |
| Methane (air emission) | kg | **1.2E-04** |  |  |  |  |  | 1.2E-04 |  |  |  |  |  |
| Nitrous oxide (air emission) | kg | **1.7E-04** |  |  |  |  |  | 1.7E-04 |  |  |  |  |  |
| Phosphorus (water emission) | kg | **5.5E-04** |  |  |  |  |  |  |  |  |  |  | 5.5E-04 |
| Ammonia (water emission) | kg | **0.0077** |  |  |  |  |  |  |  |  |  |  | 0.0077 |
| Suspended solids (water emission) | kg | **0.021** |  |  |  |  |  |  |  |  |  |  | 0.021 |
| Dissolved solids (water emission) | kg | **0.0052** |  |  |  |  |  |  |  |  |  |  | 0.0052 |
| WWT effluent | m^3^ | **0.85** |  |  |  |  |  |  |  |  |  |  | 0.85 |
| ^a^ Sewer pipe and WWTP infrastructure and installation/removal not displayed in table. | | | | | | | | | | | | | |

1. Inputs for wastewater infrastructure during wastewater collection, treatment and discharge

The Tables S9-S13 describe the material inputs for wastewater collection piping, waste water treatment units at unit process level, and on-site piping for wastewater treatment and discharge.

Table S9. Infrastructure requirements for sewer collection system piping (per m^3^ sewer collected from consumer)

|  | **Pipe Material** | | | | |  |
| --- | --- | --- | --- | --- | --- | --- |
| Diameter (in) | *PVC (m)* | *Vitrified Clay (m)* | *Concrete (m)* | *Reinforced Concrete (m)* | *Cement-Lined Ductile Iron (m)* | *Earthworks (m^3^)* |
| 8 | 1.8E-05 | 2.4E-05 | 8.9E-06 | 2.4E-07 | 5.4E-07 | 7.8E-05 |
| 10 | 3.6E-08 | 2.5E-06 | 4.6E-07 | 1.9E-08 | 3.2E-08 | 5.1E-06 |
| 12 | 4.7E-06 | 2.6E-05 | 4.6E-05 | 3.8E-06 | 6.9E-07 | 1.5E-04 |
| 15 | 1.1E-06 | 6.3E-06 | 2.9E-06 | 5.7E-07 | 0 | 2.2E-05 |
| 16 | 0 | 0 | 0 | 0 | 1.6E-07 | 3.4E-07 |
| 18 | 9.6E-07 | 5.1E-06 | 2.8E-06 | 6.3E-07 | 1.1E-07 | 2.2E-05 |
| 20 | 0 | 6.0E-07 | 0 | 0 | 4.4E-08 | 1.6E-06 |
| 21 | 4.3E-07 | 1.1E-06 | 9.4E-07 | 3.6E-07 | 0 | 7.1E-06 |
| 24 | 1.5E-06 | 2.7E-06 | 1.7E-06 | 4.3E-07 | 3.1E-07 | 1.8E-05 |
| 27 | 1.6E-07 | 2.0E-07 | 1.8E-07 | 2.9E-07 | 0 | 2.6E-06 |
| 30 | 8.7E-07 | 2.2E-07 | 1.7E-06 | 1.1E-06 | 3.2E-07 | 1.4E-05 |
| 33 | 0 | 8.5E-08 | 2.3E-07 | 9.7E-08 | 0 | 1.5E-06 |
| 36 | 4.3E-07 | 2.5E-07 | 6.7E-07 | 1.3E-06 | 2.4E-07 | 1.2E-05 |
| 42 | 0 | 6.7E-08 | 7.7E-07 | 6.1E-07 | 0 | 6.7E-06 |
| 48 | 0 | 0 | 4.3E-07 | 7.3E-07 | 0 | 6.2E-06 |
| 54 | 0 | 0 | 2.5E-07 | 5.1E-07 | 0 | 4.6E-06 |
| 60 | 0 | 0 | 3.8E-07 | 1.8E-06 | 0 | 1.5E-05 |
| 66 | 0 | 0 | 5.6E-08 | 5.8E-07 | 0 | 4.9E-06 |
| 72 | 0 | 0 | 2.3E-07 | 5.4E-07 | 0 | 6.6E-06 |
| 96 | 0 | 0 | 8.4E-08 | 7.8E-07 | 0 | 1.1E-05 |

Table S10. Material inputs for wastewater treatment plant treatment tanks and buildings

|  | **Per m^3^ of water treated** | | | | | |
| --- | --- | --- | --- | --- | --- | --- |
| **Life Cycle Stage** | **Concrete (kg)** | **Steel (kg)** | **HDPE (kg)** | **Earthworks (cu ft)** | **Concrete (m^3^)** | **Earthworks (m^3^)** |
| Pumping, at WWT Plant | 1.6E-05 | 3.9E-05 |  | 9.0E-05 | 6.7E-09 | 2.6E-06 |
| Screening and Grit Removal | 0.0016 | 9.8E-05 | 1.2E-06 | 9.3E-05 | 6.8E-07 | 2.6E-06 |
| Primary Sedimentation | 0.025 | 8.8E-04 | 5.8E-06 | 2.2E-05 | 1.0E-05 | 6.1E-07 |
| Aeration | 0.018 | 6.4E-04 | 0 | 8.6E-05 | 7.3E-06 | 2.4E-06 |
| Secondary Clarifiers | 0.0076 | 2.7E-04 | 0 | 5.3E-05 | 3.1E-06 | 1.5E-06 |
| Sludge Thickening and Dewatering | 0.0011 | 1.2E-04 | 1.6E-05 | 1.7E-04 | 4.7E-07 | 4.9E-06 |
| Sludge Incineration | 1.2E-05 | 5.8E-05 | 0 | 6.8E-05 | 5.1E-09 | 1.9E-06 |
| Primary Disinfection, Sodium Hypochlorite | 0 | 0 | 2.2E-06 | 0 | 0 | 0 |

Table S11. Material inputs for motors at wastewater treatment plant

|  | **Per m^3^ of water treated** | | | | |
| --- | --- | --- | --- | --- | --- |
| **Life Cycle Stage** | **Electrical steel (kg)** | **Other Steel (kg)** | **Cast Iron (kg)** | **Aluminum (kg)** | **Copper (kg)** |
| Pumping, at WWT Plant | 3.7E-06 | 7.9E-07 | 3.6E-06 | 2.1E-07 | 6.4E-07 |
| Screening and Grit Removal | 1.3E-08 | 3.6E-09 | 1.8E-08 | 3.9E-09 | 2.8E-09 |
| Primary Sedimentation | 2.4E-08 | 6.0E-09 | 1.8E-07 | 5.4E-09 | 4.6E-09 |
| Sludge Thickening and Dewatering | 2.4E-06 | 5.2E-07 | 2.6E-06 | 1.6E-07 | 4.2E-07 |
| Sludge Incineration | 9.5E-08 | 2.1E-08 | 1.8E-07 | 6.4E-09 | 1.7E-08 |
| Aeration | 1.3E-05 | 2.8E-06 | 1.3E-05 | 7.6E-07 | 2.3E-06 |
| Primary disinfection | 0 | 0 | 0 | 0 | 0 |
| Release wastewater effluent | 0 | 0 | 0 | 0 | 0 |
| Secondary Clarifiers | 5.1E-08 | 1.4E-08 | 3.2E-07 | 1.4E-08 | 1.1E-08 |

Table S12. Material inputs for pumps at wastewater treatment plant

|  | **Per m^3^ of water treated** | |
| --- | --- | --- |
| **Life Cycle Stage** | **Cast iron (kg)** | **Stainless 18/8 coil (kg)** |
| Pumping, at WWT Plant | 2.4E-05 | 2.2E-06 |
| Screening and Grit Removal | 5.5E-08 | 8.4E-09 |
| Primary Sedimentation | 1.3E-07 | 9.1E-08 |
| Sludge Thickening and Dewatering | 2.8E-07 | 1.9E-07 |
| Sludge Incineration | 2.2E-07 | 5.7E-08 |
| Aeration | 0 | 0 |
| Primary disinfection | 0 | 0 |
| Release wastewater effluent | 0 | 0 |
| Secondary Clarifiers | 4.6E-06 | 7.2E-07 |

Table S13. Material inputs of pipes for wastewater treatment and discharge

|  |  | **Length (m/m^3^)** | | **Earth Excavated (m^3^/m^3^)** | | **Earthworks** | **Earthworks** |
| --- | --- | --- | --- | --- | --- | --- | --- |
| **Life Cycle Stage** | **Diameter (in)** | **Ductile Iron** | **Reinforced Concrete** | **Ductile Iron** | **Reinforced Concrete** | **Total** | **Total for Piping by Life Cycle Stage** |
| Screening and Grit Removal | 48 | 0 | 7.9E-09 | 0 | 4.2E-08 | 4.2E-08 | 3.2E-07 |
|  | 72 | 0 | 1.7E-09 | 0 | 1.5E-08 | 1.5E-08 |  |
|  | 90 | 0 | 1.3E-09 | 0 | 1.5E-08 | 1.5E-08 |  |
|  | 96 | 0 | 2.0E-08 | 0 | 2.5E-07 | 2.5E-07 |  |
| Primary Sedimentation | 8 | 5.8E-08 | 0 | 8.9E-08 | 0 | 8.9E-08 | 3.7E-07 |
|  | 16 | 0 | 1.3E-08 | 0 | 2.7E-08 | 2.7E-08 |  |
|  | 96 | 0 | 2.0E-08 | 0 | 2.5E-07 | 2.5E-07 |  |
| Aeration | 8 | 6.9E-09 | 0 | 1.1E-08 | 0 | 1.1E-08 | 2.9E-07 |
|  | 10 | 1.4E-08 | 0 | 2.4E-08 | 0 | 2.4E-08 |  |
|  | 12 | 1.9E-08 | 0 | 3.4E-08 | 0 | 3.4E-08 |  |
|  | 120 | 0 | 1.3E-08 | 0 | 2.2E-07 | 2.2E-07 |  |
| Sludge Thickening and Dewatering | 6 | 7.3E-09 | 0 | 1.0E-08 | 0 | 1.0E-08 | 5.8E-07 |
|  | 8 | 1.1E-07 | 0 | 1.7E-07 | 0 | 1.7E-07 |  |
|  | 10 | 4.1E-08 | 0 | 6.9E-08 | 0 | 6.9E-08 |  |
|  | 12 | 2.6E-09 | 0 | 4.7E-09 | 0 | 4.7E-09 |  |
|  | 16 | 6.4E-08 | 0 | 1.3E-07 | 0 | 1.3E-07 |  |
|  | 20 | 4.3E-08 | 0 | 1.1E-07 | 0 | 1.1E-07 |  |
|  | 48 | 1.6E-08 | 0 | 8.5E-08 | 0 | 8.5E-08 |  |
| Sludge Incineration | 10 | 2.9E-08 | 0 | 4.8E-08 | 0 | 4.8E-08 | 1.6E-07 |
|  | 12 | 3.1E-08 | 0 | 5.6E-08 | 0 | 5.6E-08 |  |
|  | 16 | 2.8E-08 | 0 | 5.9E-08 | 0 | 5.9E-08 |  |
| Release of Wastewater Effluent | 120 | 0 | 1.0E-07 | 0 | 1.8E-06 | 1.8E-06 | 1.8E-06 |

1. Assumptions of lifetime for infrastructure components

Table S14. Assumptions of life cycle infrastructure components

|  | **Pipe Material** | | | | | **Motor and Pumps** | **Treatment Tanks** |
| --- | --- | --- | --- | --- | --- | --- | --- |
|  | ***PVC*** | ***Vitrified Clay*** | ***Concrete*** | ***Reinforced Concrete*** | ***Cement-Lined Ductile Iron*** |  |  |
| Lifetime (Years) | 55 | 100 | 105 | 105 | 97.5 | 25 | 100 |
| *Source: American Water Works Association. 2012. Buried No Longer: Confronting America’s Water Infrastructure Challenge.* | | | | | | | |

1. **Life cycle impact categories and impact assessment methods**

Table S15 life cycle impact assessment methods and categories

| **Category** | **Methodology** | **Unit** | **Description** |
| --- | --- | --- | --- |
| Global Warming | TRACI 2.0 | kg CO_2_ eq | Represents the potential heat trapping capacity of greenhouse gases. |
| Energy Demand | ecoinvent | MJ eq | Measures the total energy use from point of extraction. |
| Fossil Depletion | ReCiPe | kg oil eq | Assesses the potential reduction of fossil fuel energy resources. |
| Acidification | TRACI 2.0 | H+ moles eq | Quantifies the potential acidifying effect of substances on their environment. |
| Eutrophication | TRACI 2.0 | kg N eq | Assesses potential impacts from excessive load of macro-nutrients to the environment. |
| Blue Water Use | Blue water footprint | m^3^ | Calculates consumptive use of fresh surface or groundwater. |
| Smog | TRACI 2.0 | kg O_3_ eq | Determines the potential formation of reactive substances (e.g. tropospheric ozone) that cause harm to human health and vegetation. |
| Ozone Depletion | TRACI 2.0 | kg CFC-11 eq | Measures potential stratospheric ozone depletion. |
| Metal Depletion | ReCiPe | kg Fe eq | Assesses the potential reduction of metal resources. |
| Human Health, Cancer, Total | TRACI 2.0 | CTU | A comparative toxic unit (CTU) for cancer characterizes the probable increase in cancer related morbidity (from inhalation or ingestion) for the total human population per unit mass of a chemical emitted. |
| Human Health, NonCancer, Total | TRACI 2.0 | CTU | A CTU for noncancer characterizes the probable increase in noncancer related morbidity (from inhalation or ingestion) for the total human population per unit mass of a chemical emitted. |
| Human Health, Criteria | TRACI 2.0 | kg PM10 eq | Assesses human exposure to elevated particulate matter less than 10 μm. |
| Ecotoxicity, Total | TRACI 2.0 | CTU | Assesses potential fate, exposure, and effect of chemicals on the environment. |

1. Life cycle assessment results and contributions of unit processes

Table S16. Life cycle assessment results (per m^3^ water delivered and subsequently treated)

| **Category** | **Unit** | **Result** |
| --- | --- | --- |
| Global Warming | kg CO_2_ eq | 1.54 |
| Energy Demand | MJ eq | 28.1 |
| Fossil Depletion | kg oil eq | 0.52 |
| Acidification | H+ moles eq | 0.63 |
| Eutrophication | kg N eq | 0.011 |
| Blue Water Use | m^3^ | 1.21 |
| Smog | kg O_3_ eq | 0.094 |
| Ozone Depletion | kg CFC-11 eq | 3.7E-08 |
| Metal Depletion | kg Fe eq | 0.046 |
| Human Health, Cancer, Total | CTU | 3.9E-11 |
| Human Health, NonCancer, Total | CTU | 4.1E-11 |
| Human Health, Criteria | kg PM10 eq | 0.0019 |
| Ecotoxicity, total | CTU | 6.9E-04 |

Table S17. Contributions of unit processes

| Life Cycle Stage | Cost | Global Warm-ing | Energy Dem-and | Fossil Deple-tion | Acidifi-cation | Eutrophi-cation | Blue Water Use | Smog | Ozone Deple-tion | Metal Deple-tion | Human Health, Cancer, Total | Human Health, NonCancer, Total | Human Health, Criteria | Ecotox-icity, total |
| --- | --- | --- | --- | --- | --- | --- | --- | --- | --- | --- | --- | --- | --- | --- |
| Source Water Acquisition | 0.00% | 0.05% | 0.04% | 0.04% | 0.02% | 0.00% | 98.59% | 0.05% | 0.18% | 0.16% | 0.19% | 0.07% | 0.04% | 0.24% |
| DWTP, Plant Energy Usage | 5.59% | 20.04% | 20.98% | 20.78% | 21.38% | 0.33% | 0.26% | 20.10% | 18.19% | 1.75% | 20.54% | 3.56% | 19.78% | 11.37% |
| DWTP, Flocculation | 4.28% | 0.94% | 0.95% | 0.85% | 1.99% | 0.04% | 0.06% | 1.11% | 3.79% | 2.43% | 2.37% | 4.22% | 3.14% | 2.10% |
| DWTP, Sedimentation | 0.30% | 0.47% | 0.17% | 0.21% | 0.20% | 5.50% | 0.00% | 0.26% | 0.37% | 0.48% | 0.14% | 0.78% | 0.28% | 0.25% |
| DWTP, Filtration | 0.13% | 0.03% | 0.02% | 0.02% | 0.01% | 0.18% | 0.00% | 0.03% | 0.07% | 0.22% | 0.08% | 0.06% | 0.03% | 0.07% |
| DWTP, Adsorption | 6.59% | 3.17% | 2.85% | 3.44% | 1.37% | 0.00% | 0.00% | 4.74% | 0.20% | 0.55% | 0.81% | 0.33% | 2.79% | 0.69% |
| DWTP, Conditioning | 3.91% | 3.99% | 3.80% | 3.09% | 3.55% | 2.18% | 0.12% | 2.91% | 11.03% | 11.12% | 5.66% | 32.38% | 5.20% | 17.49% |
| DWTP, Primary Disinfection | 0.56% | 0.18% | 0.21% | 0.16% | 0.12% | 0.01% | 0.00% | 0.15% | 0.49% | 0.55% | 1.06% | 4.20% | 0.17% | 0.37% |
| Water Distribution | 12.51% | 41.21% | 43.29% | 42.93% | 47.15% | 0.72% | 0.67% | 42.68% | 41.49% | 61.03% | 43.63% | 32.22% | 45.28% | 31.69% |
| Water System Overhead | 9.63% | 0.00% | 0.00% | 0.00% | 0.00% | 0.00% | 0.00% | 0.00% | 0.00% | 0.00% | 0.00% | 0.00% | 0.00% | 0.00% |
| Wastewater Collection | 18.83% | 0.87% | 0.80% | 0.91% | 1.04% | 0.05% | 0.00% | 3.00% | 0.52% | 0.57% | 0.67% | 0.50% | 0.66% | 0.64% |
| WWTP, In Plant Pumping | 2.18% | 3.44% | 3.65% | 3.61% | 3.72% | 0.06% | 0.04% | 3.50% | 3.17% | 0.74% | 3.61% | 0.96% | 3.45% | 2.04% |
| WWTP, Mobile Fuel Combustion | 1.05% | 0.26% | 0.21% | 0.26% | 0.43% | 0.03% | 0.00% | 1.78% | 0.08% | 0.00% | 0.19% | 0.28% | 0.20% | 0.22% |
| WWTP, Screening and Grit Removal | 0.02% | 0.07% | 0.08% | 0.08% | 0.06% | 0.00% | 0.00% | 0.12% | 0.40% | 0.71% | 0.13% | 0.17% | 0.10% | 0.23% |
| WWTP, Primary Sedimentation | 0.56% | 0.79% | 0.70% | 0.67% | 0.59% | 0.02% | 0.01% | 0.71% | 1.26% | 6.34% | 1.32% | 2.38% | 0.85% | 1.10% |
| WWTP, Secondary Clarifiers | 0.27% | 0.51% | 0.50% | 0.50% | 0.49% | 0.01% | 0.01% | 0.50% | 0.56% | 1.89% | 0.68% | 0.45% | 0.54% | 0.44% |
| WWTP, Sludge Thickening and Dewatering | 10.55% | 3.68% | 4.42% | 4.74% | 3.15% | 0.05% | 0.04% | 3.17% | 2.96% | 2.01% | 3.09% | 3.45% | 3.07% | 20.26% |
| WWTP, Aeration | 7.75% | 12.4% | 13.04% | 12.92% | 13.26% | 0.20% | 0.16% | 12.55% | 11.60% | 5.63% | 13.22% | 4.13% | 12.23% | 7.56% |
| WWTP, Sludge Incineration | 2.50% | 7.08% | 3.44% | 4.09% | 1.02% | 0.07% | 0.01% | 1.96% | 0.95% | 0.53% | 1.16% | 0.47% | 1.48% | 1.06% |
| WWTP, Primary Disinfection | 1.49% | 0.79% | 0.85% | 0.71% | 0.46% | 0.03% | 0.01% | 0.67% | 2.70% | 3.30% | 1.44% | 9.38% | 0.71% | 2.17% |
| Wastewater Effluent Discharge | 0.00% | 0.00% | 0.00% | 0.00% | 0.00% | 90.54% | 0.00% | 0.01% | 0.00% | 0.01% | 0.00% | 0.00% | 0.00% | 0.00% |
| Wastewater System Overhead | 11.31% | 0.00% | 0.00% | 0.00% | 0.00% | 0.00% | 0.00% | 0.00% | 0.00% | 0.00% | 0.00% | 0.00% | 0.00% | 0.00% |
| total | 100% | 100% | 100% | 100% | 100% | 100% | 100% | 100% | 100% | 100% | 100% | 100% | 100% | 100% |

1. Contributions of infrastructure stage at unit process level

Table S18. Contributions of infrastructure stage at unit process level

| **Life Cycle Stage** | **Unit Process** | **Global Warming** | **Energy Demand** | **Fossil Depletion** | **Acidification** | **Eutro-phication** | **Blue Water Use** | **Smog** | **Ozone Depletion** | **Metal Depletion** | **Human Health, Cancer, Total** | **Human Health, NonCancer, Total** | **Human Health, Criteria** | **Ecotoxicity, total** |
| --- | --- | --- | --- | --- | --- | --- | --- | --- | --- | --- | --- | --- | --- | --- |
| Source Water Acquisition | Source water acquisition infrastructure | 0.05% | 0.04% | 0.04% | 0.02% | 0.00% | 0.00% | 0.05% | 0.18% | 0.16% | 0.19% | 0.07% | 0.04% | 0.24% |
| Pre-Disinfection | Conditioning infrastructure | 0.02% | 0.01% | 0.01% | 0.01% | 0.00% | 0.00% | 0.02% | 0.04% | 0.16% | 0.06% | 0.03% | 0.02% | 0.05% |
|  | Adsorption infrastructure | 0.08% | 0.04% | 0.04% | 0.03% | 0.00% | 0.00% | 0.07% | 0.15% | 0.55% | 0.21% | 0.12% | 0.08% | 0.16% |
|  | Filtration infrastructure | 0.03% | 0.01% | 0.02% | 0.01% | 0.00% | 0.00% | 0.03% | 0.06% | 0.20% | 0.08% | 0.04% | 0.03% | 0.06% |
|  | Lime addition infrastructure | 0.01% | 0.01% | 0.01% | 0.01% | 0.00% | 0.00% | 0.01% | 0.03% | 0.04% | 0.03% | 0.02% | 0.01% | 0.02% |
|  | Sedimentation infrastructure | 0.01% | 0.01% | 0.01% | 0.01% | 0.00% | 0.00% | 0.03% | 0.06% | 0.01% | 0.03% | 0.01% | 0.01% | 0.06% |
|  | Flocculation infrastructure | 0.04% | 0.02% | 0.03% | 0.02% | 0.00% | 0.00% | 0.04% | 0.08% | 0.28% | 0.11% | 0.06% | 0.04% | 0.10% |
| Primary Disinfection | Primary disinfection infrastructure | 0.00% | 0.00% | 0.00% | 0.00% | 0.00% | 0.00% | 0.00% | 0.00% | 0.01% | 0.00% | 0.00% | 0.00% | 0.00% |
| Water Distribution | Pipe Network | 0.71% | 0.91% | 1.11% | 0.57% | 0.03% | 0.00% | 1.57% | 0.45% | 32.95% | 0.29% | 0.08% | 0.40% | 2.51% |
|  | Water Storage | 0.01% | 0.01% | 0.01% | 0.01% | 0.00% | 0.00% | 0.01% | 0.03% | 0.50% | 0.05% | 0.08% | 0.03% | 0.04% |
|  | Valves | 0.28% | 0.25% | 0.27% | 0.14% | 0.01% | 0.00% | 0.24% | 0.61% | 16.52% | 1.41% | 2.58% | 0.83% | 1.27% |
|  | Pumps | 0.01% | 0.01% | 0.01% | 0.00% | 0.00% | 0.00% | 0.01% | 0.02% | 0.31% | 0.06% | 0.01% | 0.03% | 0.06% |
|  | Motors | 0.01% | 0.01% | 0.01% | 0.01% | 0.00% | 0.00% | 0.01% | 0.03% | 0.76% | 0.08% | 3.90% | 0.04% | 0.36% |
| Wastewater Treatment | Wastewater Collection Pipe Network | 0.16% | 0.15% | 0.17% | 0.15% | 0.01% | 0.00% | 0.36% | 0.13% | 0.53% | 0.09% | 0.04% | 0.13% | 0.14% |
|  | Pumping, at WWT Plant | 0.01% | 0.01% | 0.01% | 0.01% | 0.00% | 0.00% | 0.01% | 0.02% | 0.44% | 0.05% | 0.34% | 0.03% | 0.07% |
|  | Mobile Fuel Combustion at WWT Plant | 0.00% | 0.00% | 0.00% | 0.00% | 0.00% | 0.00% | 0.00% | 0.00% | 0.00% | 0.00% | 0.00% | 0.00% | 0.00% |
|  | Screening and Grit Removal | 0.02% | 0.01% | 0.02% | 0.01% | 0.00% | 0.00% | 0.02% | 0.05% | 0.66% | 0.07% | 0.12% | 0.04% | 0.06% |
|  | Primary Sedimentation | 0.27% | 0.14% | 0.15% | 0.10% | 0.01% | 0.00% | 0.22% | 0.54% | 5.95% | 0.75% | 1.21% | 0.35% | 0.62% |
|  | Secondary Clarifiers | 0.08% | 0.04% | 0.05% | 0.03% | 0.00% | 0.00% | 0.07% | 0.17% | 1.86% | 0.23% | 0.38% | 0.11% | 0.20% |
|  | Sludge Thickening and Dewatering | 0.02% | 0.02% | 0.02% | 0.01% | 0.00% | 0.00% | 0.02% | 0.05% | 0.86% | 0.08% | 0.33% | 0.05% | 0.09% |
|  | Aeration | 0.20% | 0.10% | 0.11% | 0.07% | 0.00% | 0.00% | 0.16% | 0.40% | 4.56% | 0.56% | 1.93% | 0.26% | 0.55% |
|  | Sludge Incineration | 0.01% | 0.01% | 0.01% | 0.00% | 0.00% | 0.00% | 0.01% | 0.02% | 0.40% | 0.03% | 0.07% | 0.02% | 0.03% |
|  | Primary Disinfection, Sodium Hypochlorite | 0.00% | 0.00% | 0.00% | 0.00% | 0.00% | 0.00% | 0.00% | 0.00% | 0.00% | 0.00% | 0.00% | 0.00% | 0.00% |
|  | Piping for Release of Wastewater Effluent | 0.00% | 0.00% | 0.00% | 0.00% | 0.00% | 0.00% | 0.01% | 0.00% | 0.01% | 0.00% | 0.00% | 0.00% | 0.00% |
|  | **Total** | **2.07%** | **1.81%** | **2.10%** | **1.22%** | **0.06%** | **0.03%** | **2.96%** | **3.10%** | **67.71%** | **4.46%** | **11.44%** | **2.55%** | **6.69%** |

1. Top contributing unit process and pollutant for each impact category

Table S19. Top contributing pollutants for each impact category

| **Impact category** | **Top contributor** | **Contribution percentage** |
| --- | --- | --- |
| Global Warming | carbon dioxide, fossil | 91.13% |
| Energy Demand | coal, bituminous | 59.34% |
| Fossil Depletion | coal, bituminous | 76.99% |
| Acidification | sulfur dioxide | 64.57% |
| Eutrophication | nitrogen | 54.17% |
| Smog | nitrogen oxides | 92.67% |
| Ozone Depletion | ethane | 71.05% |
| Metal Depletion | iron | 47.00% |
| Human Health, Cancer, Total | dioxins | 40.13% |
| Human Health, NonCancer, Total | carbon disulfide | 83.29% |
| Human Health, Criteria | sulfur dioxide | 73.45% |
| Ecotoxicity, total | cyanide | 20.53% |

Table S20. Top contributing processes for each impact category

| **Impact category** | **Top contributor** | **Contribution percentage** |
| --- | --- | --- |
| Global Warming | electricity, bituminous coal, at power plant | 54.65% |
| Energy Demand | electricity, bituminous coal, at power plant | 39.53% |
| Fossil Depletion | electricity, bituminous coal, at power plant | 62.82% |
| Acidification | electricity, bituminous coal, at power plant | 52.37% |
| Eutrophication | release of wastewater effluent | 53.86% |
| Smog | electricity, bituminous coal, at power plant | 73.23% |
| Ozone Depletion | crude oil, at production onshore | 9.77% |
| Metal Depletion | iron ore, 46% Fe, at mine | 9.17% |
| Human Health, Cancer, Total | electricity, bituminous coal, at power plant | 15.49% |
| Human Health, NonCancer, Total | copper concentrate, at beneficiation | 32.40% |
| Human Health, Criteria | electricity, bituminous coal, at power plant | 54.86% |
| Ecotoxicity, total | acrylic acid, at plant | 7.59% |

1. Electricity mixes for US average and Cincinnati region

Table S21. U.S. electrical grid fuel profiles^[[7]](#footnote-7)^

| **Electricity source** | **U.S. Average** | **RFCW NERC Region** |
| --- | --- | --- |
| Bituminous coal | 46.24% | 77.06% |
| Lignite coal | 1.96% | 0% |
| Natural gas | 21.43% | 2.41% |
| Distillate oil | 0.18% | 0.14% |
| Residual oil | 0.57% | 0.0024% |
| Biomass | 1.33% | 0.48% |
| Nuclear | 19.57% | 18.24% |
| Hydro | 6.03% | 0.62% |
| Wind | 1.34% | 0.20% |
| Solar | 0.021% | 0% |
| Geothermal | 0.36% | 0% |
| MSW, non-biogenic | 0.15% | 0.028% |
| Petroleum coke | 0.35% | 0.20% |
| Petroleum waste oil | 0.022% | 0.0014% |
| Tire derived fuel | 0.030% | 0.0083% |
| Other fuels | 0.072% | 0.060% |
| Other gases | 0.28% | 0.54% |

1. Sensitivity analysis results

Table S22 shows the 16 input parameters for sensitivity analyses, and whether LCA or LCC results were affected. For example, changing the quantity of chlorine used at the plant would change both environmental and economic impacts. In contrast, varying the carbon content of incinerated sludge will only change the LCA results. Although the infrastructure life time can influence both LCA and LCC results, this study did not quantify the impacts of infrastructure life time on LCC results due to data limitation (see section 2.2).

Table S22 Input parameters for the sensitivity analysis

| **Parameter** | **Ranges** | **LCA** | **LCC** |
| --- | --- | --- | --- |
| Chlorine usage | Minimum, maximum, and average values obtained from GCWW | Yes | Yes |
| Lime consumption | Minimum, maximum, and average values obtained from GCWW | Yes | Yes |
| Alum coagulant usage | Minimum, maximum, and average values obtained from GCWW | Yes | Yes |
| Sodium hypochlorite usage during distribution | Minimum, maximum, and average values obtained from GCWW | Yes | Yes |
| Natural gas for GAC reactivation | Minimum, maximum, and average values obtained from GCWW | Yes | Yes |
| Electricity usage at DWTP | ±10% of value obtained from GCWW | Yes | Yes |
| Electricity usage during distribution | ±10% of value obtained from GCWW | Yes | Yes |
| Lifetime of DWT infrastructure | ±25 years for buildings, tanks and pipes | Yes | No |
| Lifetime of water distribution infrastructure | ±25 years for pipes; ±10 years for pumps and motors | Yes | No |
| Electricity usage at wastewater treatment plant (WWTP) | ±10% of value obtained from MSDGC | Yes | Yes |
| Electricity usage during wastewater collection | ±10% of value obtained from MSDGC | Yes | Yes |
| Electricity grid | Average U.S. grid, Reliability First Corporation West (RFCW) North American Electrical Reliability Corporation (NERC) regional grid; the electricity mix is shown in Table S21. | Yes | No |
| Sodium hypochlorite consumption | ±10% of value obtained from MSDGC | Yes | Yes |
| Carbon content of incinerated sludge | IPCC gives range of 40-50% carbon content of dry sludge. Baseline modeled = 45%, minimum = 40%, maximum = 50% | Yes | No |
| Lifetime of wastewater collection infrastructure | ±25 years for pipes; ±10 years for pumps and motors | Yes | No |
| Lifetime of WWTP infrastructure | ±25 years for buildings, tanks and pipes | Yes | No |
| Electricity unit cost | ±20% of value obtained from GCWW | No | Yes |

Table S23. Sensitivity of LCA impacts of water and wastewater systems in Cincinnati due to various operational factors

|  | **Natural Gas Usage (DWT)** | | **Chlorine Usage (DWT)** | | **Alum Usage (DWT)** | | **Lime Usage (DWT)** | | **Sodium Hypochlorite Usage (DWT + WWT)** | |
| --- | --- | --- | --- | --- | --- | --- | --- | --- | --- | --- |
|  | **min** | **max** | **min** | **max** | **min** | **max** | **min** | **max** | **min** | **max** |
| Global Warming | -0.26% | 0.53% | 0.00% | 0.13% | -0.26% | 0.26% | -0.26% | 0.26% | -0.10% | 0.23% |
| Energy Demand | -0.20% | 0.50% | -0.10% | 0.10% | -0.30% | 0.30% | -0.10% | 0.10% | -0.20% | 0.46% |
| Fossil Depletion | -0.20% | 0.60% | 0.00% | 0.10% | -0.30% | 0.30% | -0.10% | 0.10% | -0.20% | 0.46% |
| Acidification | 0.00% | 0.10% | 0.00% | 0.00% | -0.60% | 0.60% | -0.10% | 0.10% | -0.10% | 0.23% |
| Eutrophication | -0.10% | 0.00% | 0.00% | 0.00% | -0.10% | 0.00% | 0.00% | 0.00% | 0.00% | 0.00% |
| Blue Water Use | 0.00% | 0.00% | 0.00% | 0.00% | 0.00% | 0.00% | 0.00% | 0.00% | -0.25% | 0.65% |
| Smog | -0.10% | 0.20% | 0.00% | 0.10% | -0.30% | 0.30% | -0.10% | 0.10% | -0.20% | 0.46% |
| Ozone Depletion | 0.00% | 0.00% | -0.10% | 0.20% | -1.20% | 1.20% | -0.20% | 0.20% | -1.26% | 1.79% |
| Metal Depletion | 0.00% | 0.40% | -0.20% | 0.20% | -0.30% | 1.00% | 0.00% | 0.00% | -1.76% | 2.69% |
| Human Health, Cancer | 0.00% | 0.10% | -0.30% | 0.50% | -0.70% | 0.70% | 0.00% | 0.00% | -0.63% | 0.90% |
| Human Health, NonCancer | 0.00% | 0.00% | -1.10% | 1.90% | -1.40% | 1.40% | 0.00% | 0.00% | -4.99% | 6.84% |
| Human Health, Criteria | -0.20% | 0.30% | 0.00% | 0.10% | -1.30% | 0.80% | -0.20% | 0.30% | 0.00% | 0.00% |
| Ecotoxicity | -0.50% | 0.00% | -0.10% | 0.20% | 0.00% | 0.00% | 0.00% | 0.00% | 0.00% | 0.00% |

Table S24. Sensitivity of LCA impacts of water and wastewater systems in Cincinnati to various infrastructure lifetime

|  | **DWTP Lifetime** | | **Distribution System Lifetime** | | **WWTP Lifetime** | | **Collection System Lifetime** | |
| --- | --- | --- | --- | --- | --- | --- | --- | --- |
|  | **min** | **max** | **min** | **max** | **min** | **max** | **min** | **max** |
| Global Warming | 0.1% | 0.0% | 0.3% | -0.2% | 0.2% | -0.1% | 0.0% | 0.0% |
| Energy Demand | 0.0% | 0.0% | 0.4% | -0.2% | 0.1% | -0.1% | 0.1% | 0.0% |
| Fossil Depletion | 0.1% | 0.0% | 0.5% | -0.3% | 0.1% | -0.1% | 0.1% | 0.0% |
| Acidification | 0.0% | 0.0% | 0.3% | -0.1% | 0.1% | 0.0% | 0.1% | 0.0% |
| Eutrophication | 0.0% | 0.0% | 0.0% | 0.0% | 0.0% | 0.0% | 0.0% | 0.0% |
| Blue Water Use | 0.0% | 0.0% | 0.0% | 0.0% | 0.0% | 0.0% | 0.0% | 0.0% |
| Smog | 0.1% | -0.1% | 0.6% | -0.4% | 0.2% | -0.1% | 0.1% | -0.1% |
| Ozone Depletion | 0.2% | -0.1% | 0.4% | -0.2% | 0.4% | -0.2% | 0.0% | 0.0% |
| Metal Depletion | 0.5% | -0.3% | 17.4% | -10.3% | 5.0% | -3.0% | 0.2% | -0.1% |
| Human Health, Cancer | 0.2% | -0.1% | 0.7% | -0.4% | 0.6% | -0.4% | 0.0% | 0.0% |
| Human Health, NonCancer | 0.1% | -0.1% | 3.5% | -1.7% | 1.5% | -0.9% | 0.0% | 0.0% |
| Human Health, Criteria | 0.1% | 0.0% | 0.5% | -0.3% | 0.3% | -0.2% | 0.0% | 0.0% |
| Ecotoxicity | 0.2% | -0.1% | 1.6% | -0.9% | 0.6% | -0.3% | 0.0% | 0.0% |

Figure S3 Sensitivity of costs to various factors including chlorine usage, chlorine unit cost, electricity consumption, and electricity unit cost.

Figure S4 Sensitivity of LCA impacts to electricity usage.

1. Comparison with previous studies

Table S25. Scope comparison with previous studies

|  | This study | Lemos et al. | Lundie et al. | Amores et al. | Mahgoub et al. | Friedrich et al. | Lassaux et al. |
| --- | --- | --- | --- | --- | --- | --- | --- |
| System boundary | Water abstraction, treatment, and distribution; and wastewater collection, treatment, and disposal | Water abstraction, treatment, and distribution; and wastewater collection, treatment, and disposal; and water administration | Water abstraction, treatment, and distribution; and wastewater collection, treatment, and disposal; and water administration | Water abstraction, treatment, and distribution; and wastewater collection, treatment, and disposal | Water abstraction, treatment, and distribution; and wastewater collection, treatment, and disposal | Water abstraction, treatment, and distribution; and wastewater collection, treatment, and disposal | Water abstraction, treatment, and distribution; and wastewater collection, treatment, and disposal |
| Infrastructure components | Both infrastructure and operation phases for all stages were included | Only operation phase was included | Both infrastructure and operation phases for all stages were included | Infrastructure phase for water abstraction, water distribution and sewerage collection stages; and operation phase for all stages were included | Only operation phase was included | Both infrastructure and operation phases for all stages were included | Both infrastructure and operation phases for all stages were included |
| Geographical representation | Cincinnati, US | Aveiro, Portugal | Sydney, Australia | Tarragona, Spain | Alexandria City, Egypt | Durban,  South African | Walloon Region, Belgium |
| Year representation | 2011 | 2008 | N/A | N/A | N/A | 2004-2006 | 2000 |
| Life cycle impact assessment method | TRACI v2.0 and Recipe 2008 | Recipe 2008 |  | CML2000 | Eco-indicator 99 | CML2000 | Eco-indicator 99 and CML2000 |

Table S26. Electricity comparison with previous studies (kWh/m3)

|  | Water Acquisition and Treatment (n=23) | Drinking Water Distribution (n = 19) | Water Acquisition, Treatment and Distribution (n = 23) | Wastewater Collection (n=10) | Wastewater Treatment and Discharge (n=14) | Wastewater Collection , Treatment and Discharge (n=14) |
| --- | --- | --- | --- | --- | --- | --- |
| Cincinnati Study (This Study) | 0.27 | 0.52 | 0.79 | 0.01 | 0.45 | 0.46 |
| 1996 EPRI Report | 0.055 | 0.31 | 0.37 | NA | NA | NA |
| Massachusetts Dept. of Environmental Protect | 0.40 | N/A | N/A | NA | NA | NA |
| Energy Center of Wisconsin | 0.50 | N/A | N/A | NA | NA | NA |
| WaterRF US Study | 0.39 | 0.070 | 0.50 | NA | NA | NA |
| U.S. Geological Survey | 0.21 | 0.30 | 0.51 | NA | NA | NA |
| Iowa Study | 0.63 | 0.10 | 0.73 | NA | NA | NA |
| EPRI 2013 (Surface) | NA | NA | 0.42 | NA | NA | NA |
| deMonsabert et al. 2008 | 0.37 | 0.26 | 0.63 | NA | NA | NA |
| Maas 2009 | 0.41 | 0.17 | 0.58 | NA | NA | NA |
| deMonsabert and Liner 1998 | NA | NA | 0.11-0.44 | NA | NA | NA |
| Amores et al. 2013 | 0.55 | 0.29 | 0.85 | NA | 1.09 | 1.09 |
| Lassaux et al. 2007 | 0.2145 | 0.1755 | 0.39 | NA | 0.31 | 0.31 |
| Burton 1996 (from Arpke and Hutzler 2006) | 0.37 | NA | NA | NA | NA | NA |
| Jeong et al. 2015 | NA | NA | 0.615 | NA | NA | NA |
| Lundie et al. 2004 | 0.086 | 0.28 | 0.37 | 0.060 | 0.41 | 0.47 |
| Lemos et al. 2013 | 0.64 | 0.15 | 0.79 | 0.21 | 0.87 | 1.08 |
| Barjoveanu et al. 2013 | 0.040 | 0.27 | 0.31 | 0.040 | 0.17 | 0.21 |
| Venkatesh and Brattebo, 2001 | 0.23 | 0.18 | 0.41 | 0.060 | 0.75 | 0.81 |
| Friedrich et al. 2009 | 0.090 | 0.10 | 0.19 | 0.14 | 0.44 | 0.58 |
| Arpke and Hutzler, 2006, low | 0.34 | 0.11 | 0.45 | NA | 0.21 | 0.21 |
| Arpke and Hutzler, 2006, high | 0.37 | 0.44 | 0.81 | NA | 0.77 | 0.77 |
| Venkatesh et al. 2014, Turin | 0.40 | 0.35 | 0.75 | 0.070 | 0.95 | 1.02 |
| Venkatesh et al. 2014, Toronto | 0.41 | 0.42 | 0.83 | 0.15 | 1.40 | 1.55 |
| Venkatesh et al. 2014, Oslo | 0.30 | 0.20 | 0.50 | 0.10 | 1.30 | 1.40 |
| Venkatesh et al. 2014, Nantes | 0.65 | 0.35 | 1.00 | 0.25 | 0.85 | 1.10 |

1. Brown, Beecher, and Carpenter. Calculator Tool for Determining Greenhouse Gas Emissions for Biosolids Processing and End Use. Environmental Science and Technology. 2010, 44 (24), pp 9509–9515. [↑](#footnote-ref-1)
2. Foley, J. and P. Lant. Direct Methane and Nitrous Oxide Emissions from Full-Scale Wastewater Treatment Systems. Research by Advanced Water Care Management Center, The University of Queensland Australia for Water Services Association of Australia, http://www.wsaa.asn.au. [↑](#footnote-ref-2)
3. Monteith, Sahely, MacLean, and Bagley. A Rational Procedure for Estimation of Greenhouse-Gas Emissions from Municipal Wastewater Treatment Plants. *Water Environment Research;* Jul/Aug 2005; 77, 4; Water Resources Abstracts pg. 390. [↑](#footnote-ref-3)
4. Intergovernmental Panel on Climate Change. Guidelines for National Greenhouse Gas Inventories Volume 5: Waste. Intergovernmental Panel on Climate Change. 2006. Available at http://www.ipcc-nggip.iges.or.jp/public/2006gl/index.html [↑](#footnote-ref-4)
5. The Biosolids Emissions Assessment Model (BEAM): A Method for Determining Greenhouse Gas Emissions from Canadian Biosolids Management Practices (2009) Prepared by SYLVIS for Canadian Council of Ministers of the Environment. [↑](#footnote-ref-5)
6. National Renewable Energy Lab. US LCI Database. See: http://www.nrel.gov/lci/database/default.asp. [↑](#footnote-ref-6)
7. eGRID 2008 (Emissions and Generation Resource Integrated Database). U.S. EPA. (www.epa.gov/cleanenergy/egrid). [↑](#footnote-ref-7)
